# Levetiracetam Mechanisms of Action: From Molecules to Systems

**DOI:** 10.3390/ph15040475

**Published:** 2022-04-13

**Authors:** Itzel Jatziri Contreras-García, Noemí Cárdenas-Rodríguez, Antonio Romo-Mancillas, Cindy Bandala, Sergio R. Zamudio, Saúl Gómez-Manzo, Beatriz Hernández-Ochoa, Julieta Griselda Mendoza-Torreblanca, Luz Adriana Pichardo-Macías

**Affiliations:** 1 Laboratorio de Fisiología, Escuela Militar de Graduados de Sanidad, Ciudad de México 11200, Mexico; jatziri1984@hotmail.com; 2 Laboratorio de Neurociencias, Subdirección de Medicina Experimental, Instituto Nacional de Pediatría, Ciudad de México 04530, Mexico; noemicr2001@yahoo.com.mx; 3 Laboratorio de Diseño Asistido por Computadora y Síntesis de Fármacos, Facultad de Química, Universidad Autónoma de Querétaro, Centro Universitario, Querétaro 76010, Mexico; romtono@comunidad.unam.mx; 4 Neurociencia Básica, Instituto Nacional de Rehabilitación LGII, Secretaría de Salud, Ciudad de México 14389, Mexico; cindimiel@hotmail.com; 5 Escuela Superior de Medicina, Instituto Politécnico Nacional, Ciudad de México 11340, Mexico; 6 Departamento de Fisiología, Escuela Nacional de Ciencias Biológicas, Instituto Politécnico Nacional, Ciudad de México 07738, Mexico; zrzamudio@hotmail.com; 7 Laboratorio de Bioquímica Genética, Instituto Nacional de Pediatría, Secretaría de Salud, Ciudad de México 04530, Mexico; saulmanzo@ciencias.unam.mx; 8 Laboratorio de Inmunoquímica, Hospital Infantil de México Federico Gómez, Secretaría de Salud, Ciudad de México 06720, Mexico; beatrizhb_16@comunidad.unam.mx

**Keywords:** levetiracetam, antiepileptic drugs, SV2A, neuroprotection, neuroinflammation, GABAergic system, calcium homeostasis

## Abstract

Epilepsy is a chronic disease that affects millions of people worldwide. Antiepileptic drugs (AEDs) are used to control seizures. Even though parts of their mechanisms of action are known, there are still components that need to be studied. Therefore, the search for novel drugs, new molecular targets, and a better understanding of the mechanisms of action of existing drugs is still crucial. Levetiracetam (LEV) is an AED that has been shown to be effective in seizure control and is well-tolerable, with a novel mechanism of action through an interaction with the synaptic vesicle protein 2A (SV2A). Moreover, LEV has other molecular targets that involve calcium homeostasis, the GABAergic system, and AMPA receptors among others, that might be integrated into a single mechanism of action that could explain the antiepileptogenic, anti-inflammatory, neuroprotective, and antioxidant properties of LEV. This puts it as a possible multitarget drug with clinical applications other than for epilepsy. According to the above, the objective of this work was to carry out a comprehensive and integrative review of LEV in relation to its clinical uses, structural properties, therapeutical targets, and different molecular, genetic, and systemic action mechanisms in order to consider LEV as a candidate for drug repurposing.

## 1. Introduction

Approximately 50 million people worldwide [1] are affected by epilepsy. It is a chronic neurological disorder characterized by the presence of spontaneous and recurrent seizures caused by hyperactivity and the abnormal synchronization of neurons [2] that results in neuronal damage, inflammation, and the generation of reactive oxygen species (ROS). According to the International League Against Epilepsy (ILAE), epileptic seizures are classified as focal-onset, generalized-onset, and unknown-onset seizures (previously known as partial and generalized seizures) [3]. Hence, the choice of an appropriate drug treatment varies according to the different types of seizures.

Antiepileptic drugs (AEDs; also named antiseizure drugs) efficiently control seizures, but only in about 2/3 of people with epilepsy [4]; moreover, during the last few years it has become clear that the current way of treating epilepsy with AEDs that only controls seizures is insufficient, since it does not cover the comorbidities that epileptic patients present [5,6]. These facts have motivated the search for novel drugs with new mechanisms of action that could be more effective and have a more tolerable side effect profile. Among these, levetiracetam (LEV) stands out from other AEDs due to its novel and main mechanism of action through the interaction with the synaptic vesicle protein 2A (SV2A) [7,8,9].

LEV was approved in the early 2000s as an antiepileptic drug in both the United States and the European Union, rapidly reaching the 200,000 patient-year usage milestone by the end of 2002. LEV is a second-generation anticonvulsant drug that has demonstrated a better tolerability and an improved efficiency compared other AEDs; thus, it has gradually become a first-choice drug [10]. Even though the main use of LEV is as an AED, other clinical applications such as an anti-hyperalgesic and anti-inflammatory, and in neuropathic pain have been tested, showing promising potential [11,12,13,14]. Several analogous of LEV have been developed, notably brivaracetam (BRIV), which is currently available on the market, but many other molecules have not reached commercialization [15,16,17].

A vast number of studies have reported many other molecular targets, besides the SV2A protein, through which LEV can exert its action directly or indirectly [18,19,20]. Additionally, important evidence has shown that LEV has anti-ictogenic, antiepileptogenic, neuroprotective, anti-inflammatory, and antioxidant effects [6,21,22,23,24]. Recently, LEV has been catalogued as a multitarget drug with interesting properties that are able to address some of the current necessities in epilepsy and in other conditions [25]. According to this, the objective of this work was to perform a comprehensive and integrative review that explains the diverse aspects of LEV such as its clinical uses and effectiveness in clinical trials, structural properties, analogous, therapeutical targets, and systemic mechanisms of action in order to consider to LEV as a candidate for drug repurposing.

## 2. Methods

An exhaustive search was carried out in the following databases: Medline (via PubMed), Scopus, Science Direct, Clarivate, Google Scholar, and Clinical Trials. The bibliographic sources were selected using the following keywords alone or in combination: “Levetiracetam”, “LEV”, “drug”, “antiepileptic drug”, “racetams”, ”resistance”, “pharmaco-resistance”, “TLE”, “antiepileptogenic”, “anti-ictogenic”, “anti-inflammatory”, “temporal lobe epilepsy”, “GABA”, “GABA system”, “glutamate”, “glutamate system”, “receptor”, “pain”, “calcium”, “calcium signaling”, “calcium channels”, “SV2A”, “molecular”, ”biochemical”, “genetic”, ”genetic risk”, ”polymorphisms”, “mutation”, “mechanism”, “neuron”, ”excitotoxicity”, “oxidative markers”, “oxidative stress”, “antioxidant enzymes”, and “neuroprotection“. A total of 306 bibliographic sources, ranging from the year 1985 to February 2022 were use d. The number of articles by year of publication is listed below: 1985 (2), 1989 (1), 1992 (2), 1993 (1), 1994 (1), 1995 (2), 1996 (1), 1997 (1), 1998 (2), 1999 (3), 2000 (5), 2001 (7), 2002 (9), 2003 (7), 2004 (7), 2005 (7), 2006 (7), 2007 (9), 2008 (15), 2009 (10), 2010 (19), 2011 (12), 2012 (9), 2013 (15), 2014 (9), 2015 (13), 2016 (17), 2017 (15), 2018 (16), 2019 (21), 2020 (28), 2021 (30), and 2022 (3) of which 232 were experimental scientific articles, 62 review articles, 4 meta-analyses, 2 book chapters, and 6 web pages.

### 2.1. Inclusion Criteria

Experimental and review scientific articles indexed in the above electronic databases were included. For clinical studies: scientific publications which included individuals of all ages (neonatal, infants, teenagers, young adults, and adults), women and men treated with LEV alone or as an add-therapy and when the effect of LEV was evaluated in the human subjects. For preclinical studies, the following criteria were used: scientific publications from murine models (in vivo and in vitro) in which LEV was administered alone or in combination with other AEDs by any administration pathway (intravenous, intraperitoneal (i.p.), intragastric, intrahippocampal, via-miniosmotic bombs) and that the LEV effect was evaluated in the model studied.

In the case of epileptic animal models, we considered any model of epilepsy (pharmacological or electrical) at any stage of the disease (acute, epileptogenesis, or chronic phase). The establishment of the illness had to be demonstrated by behavioral seizures and/or electroencephalogram (EEG).

### 2.2. Exclusion Criteria

Bibliographic sources referring to normal subjects or patients with pharmacological treatment who do not have LEV within their treatment scheme. Scientific publications of experimental studies of epilepsy in rodents in the gestational stage. Scientific publications of experimental studies of epilepsy in rodents with pharmacological treatment that did not contain LEV.

## 3. Clinical Indications in Epilepsy

Nowadays, LEV is a broad-spectrum drug used alone and along with other medications for both focal-onset and generalized-onset seizures control [26]. In 1999, the U.S. Food and Drug Administration (FDA) approved the use of the oral formulation as an adjunctive therapy for the treatment of focal-onset seizures, myoclonic seizures, and generalized-onset [27]. An intravenous LEV formulation was approved in 2006 for use in patients as an adjunctive anticonvulsant therapy when oral administration was temporarily not feasible [28]. In Europe, the European Medicines Agency (EMA) approved its use in 2000 for the treatment of focal-onset seizures and focal to bilateral tonic-clonic seizures (previously known as partial seizures and partial seizures secondary generalized, respectively) as a single agent, and as an add-on treatment for focal-onset seizures, myoclonic seizures, and generalized-onset tonic-clonic seizures [29]. LEV has also shown good efficacy in patients with reflex seizures (for a review see [30]) and is probably the best new AED for the treatment of juvenile myoclonic epilepsy because of a high and sustained efficacy [31].

Of the first clinical studies that demonstrated the antiepileptic effect of LEV, one of them was conducted by Cereghino and colleagues; they found in a 38-week multicenter double-blind, randomized clinical trial that adjunctive therapy with LEV 1000 mg/day or 3000 mg/day both reduced the frequency of seizures in patients with refractory partial epilepsy. The study consisted of a 12-week, single-blind, placebo baseline period; a 4-week double-blind drug titration period; a 14-week double-blind treatment period; and an 8-week double-blind study medication withdrawal period [32,33]. Similarly, in a European randomized, double-blind, controlled trial, where LEV was evaluated for 12 weeks as an add-on therapy at doses of 1000 (500 mg twice daily) and 2000 mg/day (1000 mg twice daily) against the placebo in patients with refractory epilepsy, the results showed a reduction in seizure frequency in 22.8% of patients in the 1000 mg group and 31.6% of patients in the 2000 mg group, compared with 10.4% of patients in the placebo group [34]. Finally, a multicenter, randomized, double-blind study by Ben-Menachem and Falter compared a maintenance dose of 3000 mg LEV daily for 12 weeks with an add-on placebo in 286 patients (placebo, *n* = 105; LEV, *n* = 181) at 47 sites in patients aged 16–70 years old with difficult-to-treat focal epilepsies. From 181 LEV patients, 36 were chosen for conversion to the monotherapy phase. The results from the monotherapy group showed a reduction in focal-onset frequency and nine patients (18.4%) even remained seizure-free, which suggests that the conversion to LEV monotherapy (1500 mg twice daily) is effective and well tolerated in patients with refractory focal-onset seizures who responded to 3000 mg/day LEV as an add-on therapy [35].

Encouraging results have also been found in children and pediatric epileptic patients. Generalized-onset tonic-clonic seizures are a common type of seizure in childhood epilepsy, and treatment with LEV has shown favorable results in pediatric patients with this class of seizures [36]. A good clinical response to LEV was also seen in early-onset genetic epilepsy in both term and preterm infants [37,38]. Furthermore, LEV has been shown to be safe and effective for seizure prophylaxis in pediatric patients receiving busulfan, an antineoplastic agent that produces generalized-onset tonic-clonic seizures as a side effect [39]. In an open-label study of adjunctive LEV therapy (at 20–40 mg/kg/day) in children (aged 6–12 years) with treatment-resistant focal-onset seizures, LEV protocols (4-week baseline, 6-week titration phase an 8-week evaluation phase) were effective, safe, and well tolerated [40]. In infants and young children (1 month to <4 years) with focal-onset seizures inadequately controlled with one or two AEDs, LEV as an adjunctive therapy (40.5–50.5 mg/kg/day) was effective and well tolerated [41]. Finally, in a recent clinical trial conducted in 54 epileptic children (aged 2 to 15 years), with epileptic discharges in EEG and clinical seizures controlled with valproate, LEV (50 mg/kg) was added to the therapy to reduce EEG abnormalities. The results indicate that both groups (valproate alone and combined with LEV) reduced the severity of epileptic discharges. However, the addition of LEV normalized EEG faster [42].

Status epilepticus (SE) is an emergency condition characterized by long-term seizures in which seizures lasting more than 30 min can be fatal [43]; benzodiazepines have been used as a first-line treatment for this condition. Interestingly, in meta-analysis studies, LEV was found to be significantly effective for seizure cessation in patients with SE [44,45]. Based on this kind of evidence, LEV has been considered as a first option for second-line therapy in the treatment of SE (for a review see [46]). It is also a preferred agent due to a favorable adverse effect profile [47].

LEV is available in oral and intravenous formulations; the dose and indications as monotherapy for focal-onset seizures and focal to bilateral tonic-clonic seizures are 500 mg twice daily up to a maximum of 3 g/day. LEV (1000–3000 mg/day) has been shown to be an effective AED for both adults and children with generalized or focal-onset refractory seizures [48]. In newborns, the usual dose of LEV has been reported between 20 and 60 mg/kg/day for the treatment of neonatal seizures [49]. Moreover, in AED-naive infants and children with idiopathic generalized-onset tonic-clonic seizures, oral LEV (20–60 mg/kg/day) had a favorable 6-month treatment outcome with complete seizure control in 80.64% of patients [36].

In patients under hospitalization, treatment with LEV (500–2000 mg/day) was found to be effective in three main conditions requiring intravenous administration: SE, repetitive acute seizures, and postoperative seizures [50]. The intravenous formulation has been approved for patients 16 years of age and older; however, off-label use in children for the management of acute seizures has produced positive results [51].

Although LEV has no known serious adverse effects on organ systems, even at high-dose regimens [52], and has fewer drug-related adverse reactions than other common AEDs [53], some adverse effects have been reported (for a review see [54]). Most of them are on the central nervous system, such as somnolence, asthenia/fatigue, dizziness, hyperactivity, irritability, aggression, anxiety, depression, and rarely psychosis and suicidal thoughts [55,56,57,58,59].

On other systems, hypotension and respiratory depression were the most common adverse effects of LEV in neonates [49]. Gastrointestinal reactions such as vomiting, nausea and anorexia have also been reported [50,59,60]. Additionally, hematologic adverse effects (thrombocytopenia, eosinophilia, and rarely pancytopenia) have been reported with the use of LEV [61,62]. LEV treatment can promote infections such as pharyngitis, nasopharyngitis, and rhinitis [48,54,55]. Finally, other less frequent reactions have been reported with the use of LEV; for example, hepatic dysfunction, rhabdomyolysis, and a reduction in sperm parameters without altering sex hormone levels [63,64,65].

## 4. Other Clinical Applications

LEV has been found to have a myriad of clinical uses not only as an AED. Several studies have shown the promising potential of the anti-hyperalgesic and anti-inflammatory alternative [12]. LEV has been implicated in several studies in the management of neuropathic pain. Rossi et al. and Falah et al. reported the beneficial effects of LEV (3000 mg/day for 6 weeks) in the control of central neuropathic pain symptoms in patients with multiple sclerosis (MS) [13,14]. Brighina et al. reported that LEV (dosage of 1000 mg/day for 6 months) could improve pain in patients with migraines [66]. Hamza et al. reported the effectiveness of LEV (initially received LEV 500 mg twice a day for 2 weeks, increasing to 750 mg twice day for 2 weeks, and then to 1500 mg twice a day) in the reduction in pain in chronic lumbar radiculopathy [67].

LEV as an AED secondarily impacts on the treatment of motor disorders. In this sense, several researchers have tested this effect in other pathologies that present an abnormal motor component. Awaad et al. evaluated the effects of LEV on a vocal tic in children and adolescents with tics and Tourette syndrome. The initial starting dose of LEV was 250 mg/day. The dosage was titrated over 3 weeks to 1000 to 2000 mg/day [68]. The researchers concluded that LEV may be useful in treating tics in children and adolescents. Solaro et al. performed a multicenter, randomized, double-blind, placebo controlled, crossover study evaluating the efficacy of LEV on cerebellar signs and symptoms in (MS) patients, using a kinematic analysis. The intervention in this study consisted of three phases: first, treatment for 3 weeks with LEV or placebo; secondly, a 2-week wash-out phase; thirdly, after crossover, treatment for 3 weeks with LEV or placebo. Specifically, a digitally generated randomization algorithm with a 1:1 distribution and with no blocks was performed to allocate subjects into two groups: group 1, LEV treatment, wash-out, placebo treatment; group 2, placebo treatment, wash-out, LEV treatment. In each phase, the daily drug regimen started with a dose of 500 mg of LEV or with identical placebo capsules. The study incorporated a titration phase for LEV treatment in which the dose was increased at 3-day intervals until the maximum daily dose of 3000 mg was reached. They found that LEV (daily drug regimen started with a dose of LEV 500 mg) was effective on upper limb movement in MS patients with cerebellar signs [69]. Another preliminary study performed by Solaro et al. had similar results with a daily dose of 1500 mg LEV [70]. D’Amelio et al. reported a marked improvement on movement disorders in a 74-year-old woman with hemichorea after LEV treatment (the treatment started with 250 mg/day, seven days later the dosage was increased up to 250 mg twice a day till remission of choreic movements) [71]. Other reports have found that LEV (250 mg/day) is an option for the treatment of Sydenham chorea movement disorders in children because of the tolerability and safety profile [72,73].

Wang et al. reported that LEV (dosage of 60 mg/kg/day and a therapeutic course of 6 months) improved behavioral and cognitive functions in pediatric patients with autism spectrum disorder [74]. Deriaz et al. reported that LEV in a dose of 2 g/day over two weeks controlled de self-injurious behavior (including bruising of the limbs and biting of the tongue and lips) in a patient with pervasive developmental disorders, severe intellectual disability, and seizures, and the symptoms disappeared with no recurrence 2 years later [75]. Their results reinforced other findings related to the reduction in stress, anxiety, and panic by LEV [75,76,77]. In obsessive-compulsive disorder, LEV is recommended in a dose of 1500 mg/day [78]. Recently, Esang et al. reported evidence that LEV could reduce the risk of developing suicide-related behavior and suicidal ideation in patients who have psychiatric illness history, traumatic brain injury, or a history of substance abuse, and uncontrolled epilepsy or seizures. The LEV doses and/or duration reported in the analyzed studies were 500 mg, 1000 mg, and 3000 mg for epilepsy (*n* = 517), 500 mg/twice/day (previously 250 mg twice/day) for depression (*n* = 23), 2000 mg twice/day (previously 1000 mg/day) for alcohol dependence (*n* = 66), 1500 mg twice/day for bipolar disorder (*n* = 41), 500 mg/twice/during 2 days for a brain abnormality (*n* = 16), and 1000 mg/day during 25 days for suicidal ideations (*n* = 50) [79]. Müller et al. showed the efficacy and safety of LEV (dosage regime between 500 and 4000 mg/day for a maximum of 7 days) for outpatient alcohol detoxification; alcohol withdrawal syndrome decreased over 5 days after treatment that included LEV [80]. Furthermore, Jabbarli et al. reported through a meta-analysis a summary of the evidence on the value of AED for a glioblastoma prognosis. The authors observed that LEV could have an antitumor effect in glioblastoma and showed that perioperative treatment with LEV might improve the prognosis of glioblastoma patients associated with epilepsy (overall survival, HR = 0.83, 95%CI = 0.71–0.97, *p* = 0.02; progression-free survival, HR = 0.77, 95%CI = 0.62–0.96, *p* = 0.02). LEV was associated with longer overall survival (median: 12.8 vs. 8.77 months, *p* < 0.0001) and progression-free survival (7 vs. 4.5 months, *p* = 0.001) [81]. A randomized, double-blind, placebo-controlled study has shown LEV (500 mg/day to 3000 mg/day or placebo for 12 weeks) to be effective for tardive dyskinesia [82]. Kakisaka et al. also reported a clinical case where LEV (250 mg/day for two days) improved multiple chemical sensitivity disorder [83]. Plus, the use of LEV has been proposed for eclampsia management in pregnancy (administered as 1 g loading dose followed by 500 mg intravenously bd in 10 cases) and in Charles Bonnet syndrome (administered 500 mg twice a day for 1 day and after a dosage up to 750 mg twice for 1 day and the hallucinations completely disappeared) [84,85].

Finally, LEV presents an effect in an uncommon disorder, cyclic vomiting syndrome. This is an uncommon pathology characterized by recurrent, stereotypical episodes of vomiting separated by symptom-free intervals. Clinical studies have shown in adult patients and elderly patients that LEV (initial dose was 500 mg/day and increased in 500 mg increments, n a twice daily dosing regimen, to a median dose of 1000 mg per day) is effective for prophylaxis against cyclic vomiting syndrome episodes [86,87].

All the above show that LEV is a promising drug for the treatment of a wide variety of pathologies and several symptoms independent of epilepsy and seizures as evidenced in the previous paragraphs. LEV repositioning needs further research that not only focuses on clinical trials but also describes the molecular mechanisms of action in these pathologies.

## 5. Generalities, Chemical Structure, and Analogous

LEV (*S*-enantiomer pyrrolidine derivative of α-ethyl-2-oxo-1-pyrrolidine acetamide IUPAC, (*S*)-2-(2- oxopyrrolidin- 1-yl) butanamide) is a white to off-white crystalline powder that has a faint odor and a bitter taste. The molecular formula of LEV is C_8_H_14_N_2_O_2_, its molecular weight is 170.21, and it is water soluble. LEV has multiple advantages such as its nearly complete bioavailability and a binding protein that is less than 20% and does not affect the protein binding of other drugs [15]. In addition, LEV shows rapid and unrestricted diffusion through the blood–brain barrier (BBB) and its elimination half-life is 9 h. LEV is not metabolized in the liver, which makes it useful in patients with hepatic dysfunction and it is independent of the hepatic cytochrome P450 (CYP450) system; it is primarily excreted unchanged in the urine and 24% is metabolized into an inactive metabolite [15].

**Figure 1:**
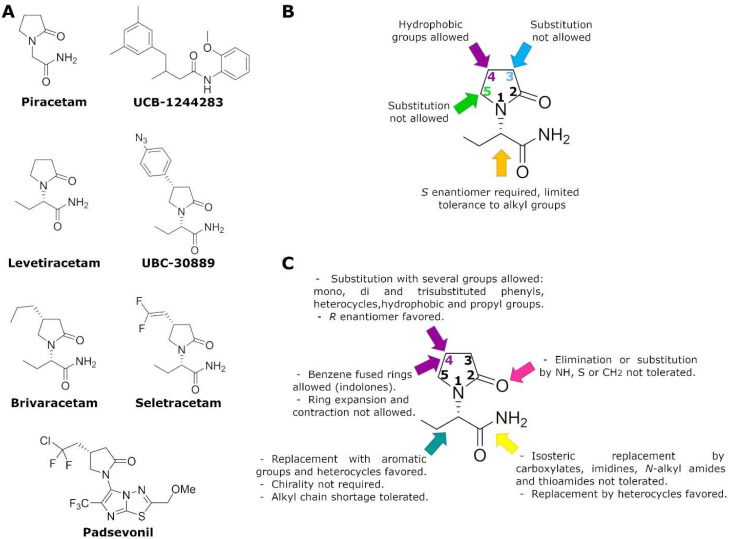
Chemical structures of racetams and molecular modifications. (**A**) Molecular structures of different SV2A ligands used in the text. (**B**) SAR map reported by Mittrapalli, 2014. (**C**) Updated SAR map according to our bibliographic search.

LEV is the *S*-enantiomer of the ethyl analog of piracetam [88] (Figure 1A). Although piracetam is a nootropic drug, LEV does not show significant nootropic effects; however, it was evaluated in epilepsy models since piracetam exhibited efficacy in the treatment of photo paroxysmal responses and myoclonus [15]. Based on the differences in the pharmacological effects between piracetam and LEV, it was not easy to establish structure–activity relationships (SAR), despite the common characteristic of 2-oxopyrrolidine ring as the central moiety. In the preliminary SAR studies, it was highlighted that the ethyl derivative of piracetam (etiracetam) had a distinctive pharmacological profile that led to its development as an anticonvulsant, while structural modifications to the 2-oxopyrrolidine ring resulted in different compounds of which only a few reached clinical stages as nootropics [89].

From the beginning of LEV development, some structurally close analogs were synthesized since they showed a high correlation between the affinity to the “LEV binding site” (LBS; see below) and protection against epileptic seizures. The first efforts to establish SAR of LEV analogs was through the modification of the carboxamide group with analogs such as carboxylic acids, nitriles, amidines, and thioamides. The *N*-substituted amides and variation of the chiral center present in this part of the molecule were also tested, highlighting the importance of the presence of the carboxamide group and the absolute configuration *S* in the substituent at position 1 of 2-oxopyrrolidine (Figure 1B). In addition, the low tolerance to substitution in positions 3 and 5, the affinity enhancement with hydrophobic substituents in position 4, and the maintenance of oxygen in position 2 underline the importance of the substitution pattern in the 2-oxopyrrolidine ring [16]. These molecular modifications were correlated to the fact that the bioactive conformation would form intramolecular hydrogen bonds between the 2-oxopyrrolidine and the carboxamide group, as well as to the lesser manifestation of severe toxic effects [16] (Figure 1B).

As part of the optimization process for anticonvulsant racetams, additional modifications were made to the 2-oxopyrrolidine ring. For example, because of the replacement of the central structure by indolones, the (S)-2-(5-chloro-2-oxoindolin-1-yl) propanamide was found, which had a better pharmacokinetic profile, fewer metabolic incidences, an affinity 100 times greater than LEV, as well as an improvement in its pharmacology on in vivo models; this molecule was patented but not further developed by UCB. The structural change in this molecule highlighted the importance of position 4, the size of the chain, and the chirality of the group present in position 1 of 2-oxopyrrolidine [90] (Figure 1C). Alternatively, molecules with a completely different central scaffold than the 2-oxopyrrolidine moiety, such as UCB-1244283 ((4-(3,5-dimethylphenyl)-*N*-(2-methoxy phenyl)-3-methylbutanamide) have been proposed. It is a positive allosteric modulator of UCB-30889 (a LEV analogue; Figure 1A), with a clear protective effect against both tonic and clonic convulsions, but only after intracerebroventricular administration because it is highly insoluble in water [91].

As a consequence of SAR studies, BRIV, the 4-(*R*)-propyl LEV analog, emerged as an promising antiepileptic candidate [16]; later, seletracetam appeared, which was a 4-(*R*)-2,2-difluoroethenyl LEV analog with a 10-fold greater affinity for SV2A than LEV [92] (Figure 1A). Seletracetam was related to the increase in short-term depression in in vitro models. Then, towards the end of the 2000s, the development of seletracetam was halted, but BRIV was successfully brought to the market [93]. Another related compound that initially showed anticonvulsant potential was phenylpiracetam, the 4-phenyl piracetam analog. It was evaluated in clinical phases for epilepsy, but it was only available in Russia as a general stimulant in 2017 [94]. Interestingly, another antiepileptic candidate of recently development was padsevonil, which integrated the pharmacophoric characteristics of ligands related to SV2A and the gamma-aminobutyric acid A receptors (GABA_A_) [95,96]. Padsevonil (Figure 1A) maintained the central nucleus of 2-oxopyrrolidine, but the insertion of hydrophobic alkyl group in position 4 and a heterocycle in position 1, resulted in both a greater affinity to SV2A than LEV and a moderately high affinity (pIC50 < 6.1) to recombinant GABA_A_ receptors [97]. Unlike LEV, padsevonil displayed a high affinity among the different isoforms of the SV2 protein (SV2A, SV2B and SV2C) and showed a greater effect in blocking seizures in various epilepsy animal models than LEV and BRIV [17]. However, by mid-2020, UCB reported that in a phase 2b study in drug-resistant focal epilepsy patients, padsevonil did not reach statistical significance for either of the primary endpoints; thus, its development program was terminated despite its good pharmacological and safety profiles [98]. Despite this setback, the discovery, design, and development of molecules with both pre and postsynaptic targets is still an interesting approach for new antiepileptic compounds.

## 6. Levetiracetam Binding Site (LBS)

Since 1995, the presence of a specific LBS that could be involved in the anticonvulsant properties of LEV has been investigated; photoaffinity labeling studies determined that UCB-30889 (the LEV analog with a 30-fold greater affinity for SV2A) was bound to a protein of ~90 kDa. This protein was only detected in brain structures but not in peripheral tissue and was abundant in synaptic plasma membranes and in the synaptic vesicle fractions [99,100,101]. This placed SV2 proteins as the primary candidates [102]. Several studies demonstrated that UCB 30889 did not bind to brain membranes and purified SVs from mice lacking SV2A, indicating that SV2A is necessary for LEV binding. Moreover, UCB 30889 bound to SV2A but not to SV2B or SV2C proteins expressed in fibroblasts, indicating that SV2A is sufficient for LEV binding [9].

**Figure 2:**
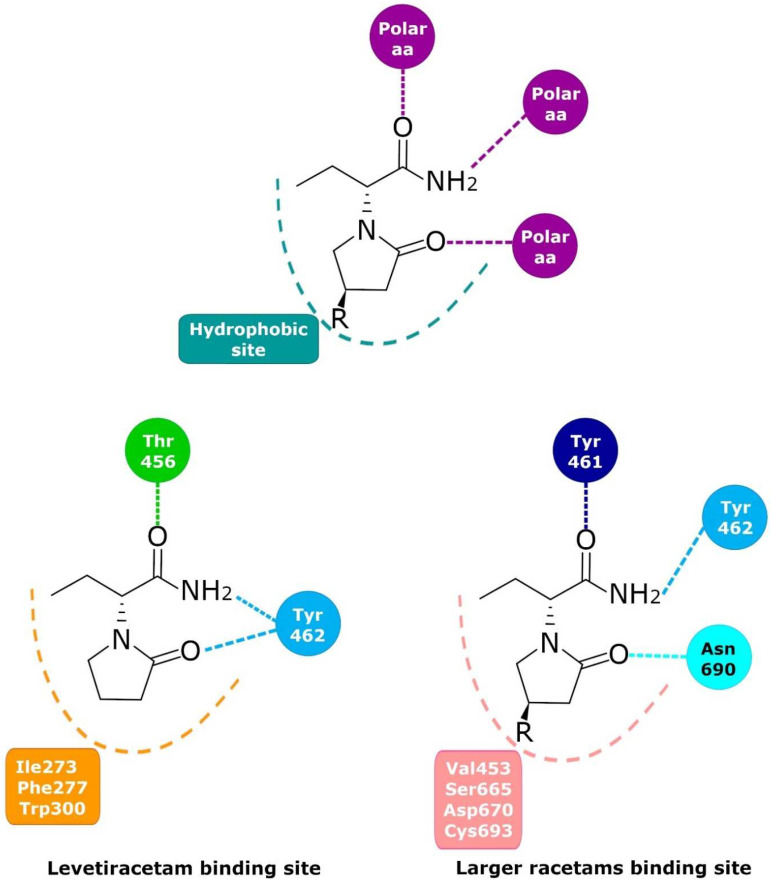
Schematic representation of the racetam binding site in SV2A found by molecular modeling. Despite having the same components, LEV binds differently than the rest of the racetams in a pocket in front of the racetam binding site that had some polar amino acids that can interact with polar groups in position 4, as in UCB-30889.

Currently, there is no doubt that LEV binds in a saturable, reversible, and stereospecific manner to SV2A in both rat and human brains; this is also true for its structural analogs [9,103]. Mutagenesis studies, molecular models, and molecular dynamics simulations have allowed important SV2A residues relevant for the LBS and its intermolecular interactions to be identified. Shi et al. found 14 residues that, when mutated, alter the binding of LEV to SV2A, they were: F277A, W300A, W300F, Y462A, K694A, G303A, F658A, V661A, I663A, W666A, N667A, S294A, M301A, and G659A [104]. Later, Lee et al. using two conformational states of the in silico model of SV2A, observed two additional residues, W454 and D670, that may contribute to LBS [105]. Finally, Correa-Basurto et al. identified additional hydrophobic and hydrogen bond interactions with T456, S665, and L689 (besides W454 and D670), which may be important for ligand recognition within the LSB and support the putative LBS observed previously [106] where the residues found by single aminoacid mutants [104] are distributed in a large volume which comprises the sites found by molecular modeling [105,106] (Figure 2). Moreover, by means of radioligand binding assays, it has been suggested that LEV and BRIV could have a different binding site or favor different conformational states of the SV2A protein [107], with the subsequent identification of the outward SV2A conformation relevant for differential binding of LEV (Ile273, Lys694, and Ser294) and BRIV (Lys694) [108]. Moreover, studies with the UCB-1244283 (SV2A positive allosteric modulator) indicated that the SV2A protein contains multiple interacting binding sites [91]. Thus, there is still a wide field of opportunities for the study of the molecular behavior of SV2A–ligand interactions (Figure 2).

## 7. Molecular Mechanism

As mentioned above, different lines of evidence suggest that SV2A modulation constitutes the primary mechanism of action of LEV. However, other targets, for example, Alpha-Amino-3-Hidroxy-5-Methyl-4-Isoxazole Propionic Acid (AMPA), noradrenaline, adenosine, and serotonin receptors, or those involved in calcium homeostasis, the gamma-aminobutyric acid (GABA) system, and intracellular pH regulation may contribute to the effects of LEV.

### 7.1. Synaptic Vesicle Protein 2A (SV2A)

**Figure 3:**
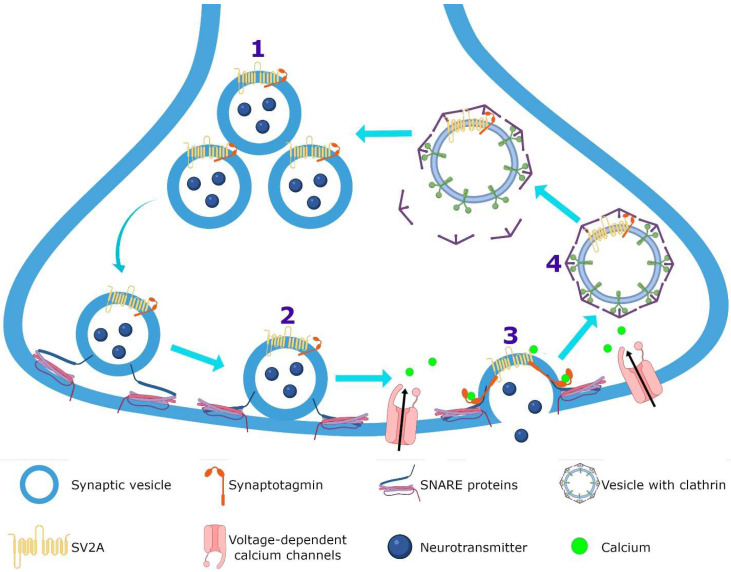
SV2A function. SV2A regulates the readily releasable pool (RRP) size (**1**) and during priming (**2**) facilitates the progression to the release-competent state, both allowing normal neurotransmission. In the exocytosis (**3**), SV2A functions as a target for residual calcium and finally, in the endocytosis (**4**), regulates the vesicle content of the calcium-sensor synaptotagmin protein.

SV2A is an integral membrane protein found in the vesicles of almost all synaptic terminals. In the synaptic vesicle cycle, several functions in both exocytosis and endocytosis processes have been attributed to SV2A. During exocytosis, the SV2A protein might function first as a target for residual Ca^2+^ (Figure 3); by means of paired pulses and the repetitive stimulation of 2 and 10 Hz in SV2A/SV2B double knockout (DKO) neurons, an increase was observed in the synaptic response (relative facilitation) with subsequent progressive depression. Such a facilitation was reversed in all frequencies by incubating DKO neurons with slow calcium buffer egtazic acid (EGTA), indicating that the difference in synaptic response was mostly due to the accumulation of residual Ca^2+^ [109,110,111]. Second, as a facilitator of the progression to the release-competent state in the vesicular priming (Figure 3). SV2A knockout (KO) or SV2A/SV2B DKO hippocampal neurons, as well as knockdown presynaptic SV2A in superior cervical ganglion neurons, resulted in a decrease in neurotransmitter release and a reduced size of the readily releasable pool (RRP) of vesicles, suggesting that SV2A maintains normal neurotransmission by regulating the RRP size [112,113,114] (Figure 3). In addition, brain tissue from SV2A KO contained a smaller proportion of the syntaxin protein, consistent with the interpretation that the loss of SV2A affects the formation of the complex of soluble NSF attachment proteins receptor (SNARE; [113,115]. Since the absence of SV2A presented a normal number of vesicles docked at the active zone in presynaptic terminal [113,115] and the mechanism of exocytosis itself was not affected (no changes were observed in any SV2A KO and SV2A/SV2B DKO hippocampal neurons when examining miniature postsynaptic currents) [109,112,113,116], but there were diminished RRP and the formation of the SNARE complexes was affected, SV2A may influence the synaptic vesicle priming step in the synaptic vesicle cycle, maintaining the availability of secretory vesicles and thus the release probability; thereby, ensuring correct neurotransmission. Third, during endocytosis, SV2A may regulate the vesicle content of the calcium-sensor synaptotagmin (SYT-1; Figure 3). Mutation in Y46 amino acid residue (an endocytosis motif) at the NH_2_ -terminus of SV2A (where SYT-1 binds to SV2A) caused a higher proportion of both SV2A and SYT-1 on the plasma membrane, indicating a reduced internalization. The Y46 residue of SV2A serves as a receptor for clathrin adaptor proteins; thus, SV2A via an interaction with both SYT-1 and clathrin adaptors may regulate the adequate trafficking of SYT-1 and, in consequence, the Ca^2+^-stimulated fusion [111,117,118].

Furthermore, there are several proposals about how LEV exerts its effect on SV2A. (1) LEV may block the effect of SV2A, inhibiting its usual role in vesicular priming, causing a decrease in RRP size and a decrease in synaptic transmission [113,114,115]. (2) Another possibility is that the binding pf LEV to SV2A could stabilize to the protein in an ideal functional conformation, resulting in potentiation or optimization of its general function [8,91,119]; then, LEV would act as improver of synaptic vesicle exocytosis. (3) LEV may enhance the role of SV2A in modulating the expression and trafficking of SYT, since LEV restores the normal levels of these protein expressions in neurons to overexpress SV2A [120,121].

**Figure 4:**
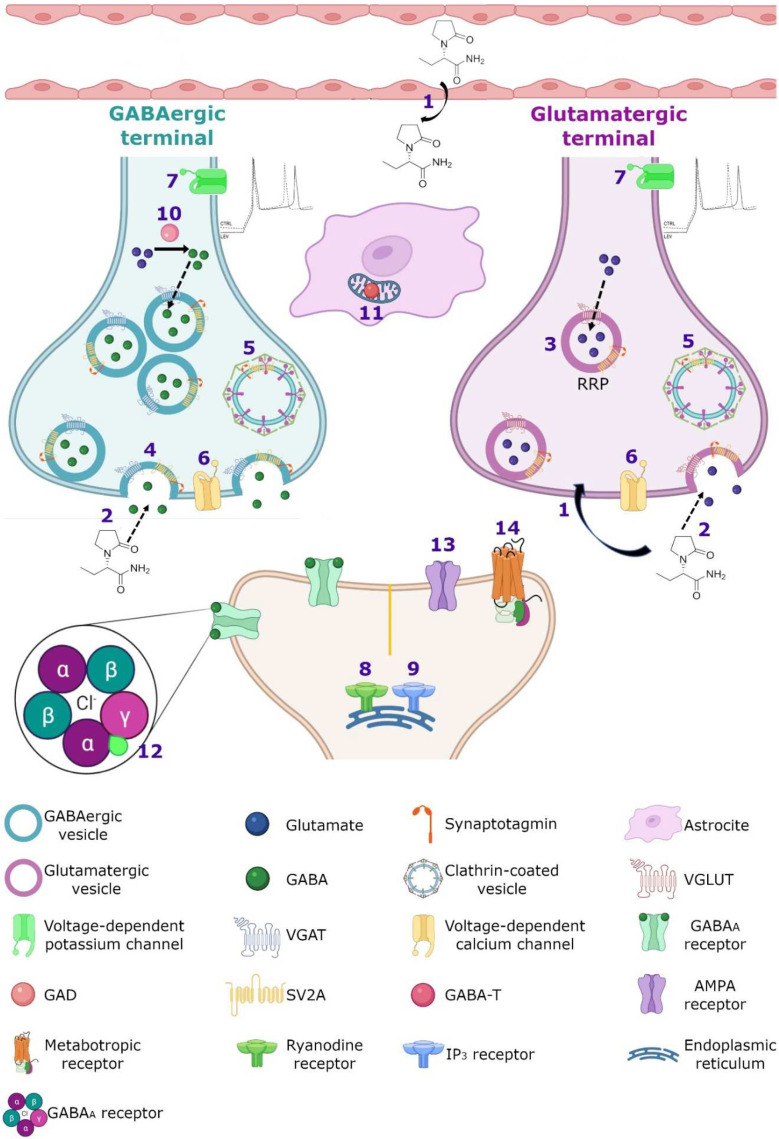
Hypothetical integrated molecular mechanisms of action of LEV. (**1**) LEV diffuses throughout the blood–brain barrier and neuron membrane or enters during (**2**) exocytosis and endocytosis processes, subsequently exerts its action by various mechanisms. (**3**) LEV could decrease the function of SV2A during vesicular priming and thus diminishes the readily releasable pool and therefore the release of neurotransmitters (purple terminal). Another possibility is that LEV stabilizes SV2A and improves its function during (**4**) exocytosis, and (**5**) endocytosis modulating the expression and traffic of synaptotagmin protein. (**6**) Moreover, it has been reported that LEV blocks the voltage-dependent calcium channels, decreasing the synaptic transmission. (**7**) LEV reduces potassium currents inducing a decrease in the repetitive action potential generation. With respect to calcium intracellular systems, LEV reduces the calcium transients of (**8**) ryanodine and (**9**) IP_3_ receptors. In the GABAergic system, LEV modulates the region-dependent (**10**) glutamic acid decarboxylase (GAD) and increases (**11**) GABA transaminase (GABA-T). In the post-synapse, LEV blocks the effect of the (**12**) GABA_A_ receptor antagonists. In the glutamatergic synapse, LEV modulates (**13**) AMPA receptors and decreases the excitatory current. (**14**) Finally, LEV interacts with noradrenaline, adenosine and serotonin receptors in post-synapse involved in pain system.

On the other hand, two hypotheses have been made to explain how LEV could reach the SV2A protein: (1) by diffusion across cellular membranes [8] and (2) by binding to SV2A during the synaptic vesicles cycle [122,123] (Figure 4). Both mechanisms are not mutually exclusive and could even be synergistic. In vitro studies performed on Caco-2 cells showed that LEV is permeable to membranes despite its low lipophilicity [124]. This concurs with the estimate of LEV permeability of 0.015 mL/min/g across the BBB, calculated in a study based on pharmacokinetic modeling. In addition, it was observed in rhesus monkeys through the SV2A PET tracer [^11^C] UCB-J that LEV enters the brain in 23 min [124]. Similar data were reported in rats, where LEV was detected in the cerebrospinal fluid, hippocampus, and frontal cortex after intraperitoneal administration of this AED [125,126,127]. Moreover, loading LEV into vesicles by sucrose stimulus (which induces the vesicular fusion), resulted in a decrease in cumulative excitatory postsynaptic currents (EPSCs) amplitudes, while the unloading of LEV with a second sucrose stimulus allowed EPSCs amplitudes to return to control levels [122]. This suggests that LEV enters the synaptic vesicle during endocytosis and exits during exocytosis. Additional experiments showed that CA1 hippocampal slices incubated with LEV reduced the amplitude of EPSCs when they were stimulated to high frequencies (80 Hz), while at slower frequencies (20 Hz) this effect was not observed [122], indicating that high frequency stimulation induces greater vesicular fusion, more entry of LEV into vesicles and, thus, a higher effect. After the administration of LEV, it diffuses throughout the BBB and reaches the brain parenchyma; then, LEV may diffuse across the neuronal membrane and/or bind to the intraluminal face of SV2A during recycling and endocytosis. Subsequently, it may be released during the exocytosis process (Figure 4).

Regarding the mechanism of the antiepileptic activity of LEV, most of the studies have focused on SV2A expression levels and/or mutations. SV2A KO mice showed severe seizures and died within 3 weeks [112]. They also presented a reduced response to LEV treatment [128]. Moreover, in the tumor and peritumoral tissues of glioma of patients with epilepsy, SV2A expression levels correlated with the clinical efficacy of LEV [129]. Moreover, treatment with LEV blocked both the development of a seizure phenotype [130] and increased the hippocampal expression of SV2A [131] in kindled mice and in primary cultures of hippocampal neurons overexpressing SV2A [121]. In other conditions, LEV treatment has also caused changes in protein expression. Inaba et al. found increases in the expression of SV2A and cAMP-triggered phosphorylation of cAMP response element binding protein (pCREB) in mice that suffered lesions in the white matter due to cerebral hypoperfusion. These animals preserved learning and memory capabilities in the Y-maze, spontaneous alternation, and novel object recognition tests [132]. Recently we found that the SV2A expression in glutamatergic terminals was a key element for the response to LEV [133]. In contrast, in the neocortex of non-epileptic rats, no significant differences were detected in SV2A protein levels in LEV-treated animals compared to controls [134]. Certainly, both an increase and decrease in SV2A expression have been reported to be associated with the presence of seizures and it could be related with an effective response to LEV treatment.

### 7.2. Calcium Homeostasis

There is significant experimental evidence showing that LEV modulates targets related with cellular Ca^2+^ which is a ubiquitous signal transduction molecule that plays a key role in the modulation of neuronal excitability and synaptic transmission. Specifically, LEV effects have been observed in voltage-gated channels and Ca^2+^ signaling. Several studies have shown that LEV can block the high-voltage activated (HVA) Ca^2+^ channels N-type, P/Q-type and L-type (Figure 4). The administration of LEV (32 µM) in CA1 of rat hippocampal slices decreased significantly the neuronal HVA Ca^2+^ currents [135]. Other studies showed a selective LEV inhibition of N-type Ca^2+^ channels in isolated striatal, neocortical, and CA1 pyramidal hippocampal neurons [136,137,138]. In addition, LEV (100 µM) provoked a partial reduction in P/Q-type HVA Ca^2+^ currents in the acutely isolated neocortical neurons [137]. Moreover, LEV inhibited Ca^2+^ entry by blocking the type Ca^2+^ L-type channels in hippocampal CA3 neurons obtained from spontaneously epileptic rats [139]. This effect was more potent than that in control neurons, suggesting that this may contribute to the antiepileptic effect of LEV [139,140]. Moreover, it has been reported that LEV elicits effects on HVA Ca^2+^ channels (presumably N-type) of superior cervical ganglion cholinergic neurons. Data showed that LEV inhibited synaptic transmission between these cells in a time-dependent manner, significantly reducing excitatory postsynaptic potential (EPSP) after a 1 h of application. Interestingly, intracellular LEV administration caused (after 4 to 5 min of exposition) rapid inhibition of the Ca^2+^ current; this is consistent with a mechanism where LEV may interact directly with HVA Ca^2+^ channels, causing a reduction in synaptic transmission [19,114]. Furthermore, the application of LEV (100 μM) in acutely isolated hippocampal CA1 neurons from rats and guinea pigs, reduced the delayer rectifier K^+^ currents by 26%, causing a decrease in the repetitive action potential generation and subsequent, leading to a slight prolongation of duration of the first action potential. Thus, LEV action may also be also related to its ability to hyperpolarize the membrane potential via K^+^ channel activation [11] (Figure 4).

On the other hand, various studies have reported that LEV is an effective inhibitor of Ca^2+^ release mediated by the two of the major systems of calcium-induced calcium release, ryanodine and inositol-3-phosphate (IP_3_) receptors (Figure 4). LEV significantly reduced the Ca^2+^ transients induced by caffeine (a ryanodine receptor activator) in cultured rat hippocampal neurons [141,142]. In addition, LEV inhibited the epileptiform effect induced by caffeine on the evoked field potentials and delayed caffeine-induced spontaneous bursting on rat hippocampal slices [141]. Moreover, LEV inhibited Ca^2+^ transients induced by bradykinin (BK; a stimulator of IP_3_ receptor) in hippocampal neurons, causing a 74% diminution in calcium release mediated by the IP_3_ receptor compared to the control [142]. In PC12 rat pheochromocytoma cells, LEV decreased in a dose-dependent manner, the increase in Ca^2+^ caused by the application of 1µM of bradykinin or 100 µM of ATP. The inhibitory effect of LEV was mainly exerted by IP_3_-triggered Ca^2+^ store depletion without reducing Ca^2+^ storage into these deposits [143]. Then, the ability of LEV to modulate ryanodine and IP_3_ receptors demonstrated another important molecular effect of this agent on a major second messenger system in neurons.

### 7.3. GABAergic System

The GABA is the main inhibitory neurotransmitter of the central nervous system. Multiple AEDs act on its GABA_A_ receptor to increase inhibition and thereby controlling the aberrant neuronal activity and seizures [144]. Regarding LEV, however, there are conflicting results concerning its effect on the GABAergic system [145]. Patients with two distinct pathologies, focal epilepsy or migraine, were treated with LEV to determine if this drug modified brain GABA levels. By means of proton magnetic resonance spectroscopy, the GABA/creatinine ratio was evaluated before and during treatment of patients with epilepsy. The data showed an increase in GABA/creatinine in the occipital lobe in responder patients (those who showed 50–100% seizure reduction), while in non-responders the results were inconclusive [146]. Meanwhile, migraine patients treated with LEV showed a decrease in headache frequency and intensity associated with a decrease in posterior cingulate cortex GABA levels [147]. These results suggest that LEV may modulate differentially GABA levels and these results agreed with several animal studies. For example, the administration of LEV before injecting the convulsive agent pilocarpine in a murine model, protected against seizures modulating different neurotransmitter release; hence, LEV reversed alterations induced by focal to bilateral tonic-clonic seizures, increasing aspartate and reducing glutamine, GABA, and glycine levels in rat hippocampus [148]. In addition, by K^+^-evoked depolarization with microdialysis technique, LEV inhibited the release of biogenic amines, GABA, and L-glutamate in the medial prefrontal cortex of control rats [149]; however, in epileptic rats treated with LEV for one week, the K^+^-evoked depolarization induced a preferential increase in GABA levels without modifying other neurotransmitters in the rat dorsal hippocampus [150]. In addition, the administration of LEV in the substantia nigra, a mainly GABAergic nucleus, showed that this anticonvulsive drug decreased the spontaneous firing of non-dopaminergic (maybe GABAergic) neurons, suggesting that the modulation of neuronal firing in GABAergic projections from the substantia nigra, could involve the activation or inhibition of neurotransmitter systems in other brain areas [148,151].

In temporal lobe epilepsy (TLE) both in animal models and patients, a run-down current elicited by GABA, which is disease progression-dependent, has been reported [152,153]; the repetitive activation of GABA_A_ receptors induces a decrease in GABAergic signaling (current) use-dependent in hippocampal and cortical neurons denominated as run-down, this desensitization of GABA_A_ receptor, could increase hyperexcitability and favor the occurrence of seizures [152,153]. In oocytes microtransplanted with ionotropic GABA_A_-receptors obtained from the resected hippocampus and temporal neocortex of patients with mesial TLE, as well as rats with pilocarpine-induced TLE, the run-down of the current evoked by GABA and the effect of LEV on this current were assessed [153,154]. In chronic epilepsy, both rats and patients showed an increase in current GABA run-down in the hippocampus and cortex and LEV had a region-dependent effect [153,154]; in the tissue of these rats, incubation with LEV did not affect the run-down current in the hippocampus, but it does attenuate it in the cortex [153]. Meanwhile, in the tissue of patients, LEV inhibited the GABA-current run-down in the hippocampus and neocortex but was ineffective in the hippocampal subiculum [154]. The authors argue that the differences could be because in the subiculum of mesial TLE patients, a switch is generated in GABA where it becomes an excitatory neurotransmitter, while in the hippocampus and neocortex GABA functions as the classic inhibitory neurotransmitter, another option to explain this data, is the differential subunit composition of GABA_A_ receptors and their phosphorylation [154].

However, there are controversial results regarding the effect of LEV in the metabolism of GABA, since on in vitro assays LEV did not alter the activity of the GABA synthesizing enzyme, glutamic acid decarboxylase (GAD) or the GABA degrading enzyme and GABA-transaminase (GABA-T); however, on in vivo studies, a decrease in GAD in striatum and an increase in the hypothalamus at high doses of the drug was observed, as well as an increase in GABA-T activity in the cortex, striatum, thalamus and cerebellum [151] (Figure 4). Moreover, LEV modified GABA turnover by reducing it in the striatum but increasing it in the cortex and hippocampus [151]. A reduction in GAD and GABA turnover in the striatum might disinhibit GABAergic striatal output pathways, augmenting the inhibition in their respective target regions and increasing the anticonvulsant effects of LEV [151]. However, the authors postulate that those differential alterations on GAD and GABA-T activity between both models and in different regions might not be directly caused by LEV, instead as a consequence to pre or postsynaptic secondary effects [151].

Another action of LEV in the GABAergic system involves the ability to reverse the inhibitory effects of the negative allosteric modulators β-carbolines and zinc on both GABA_A_ and glycine receptors, the two main ionotropic inhibitory receptor systems in the brain [145]. β-carbolines congeners can act at the benzodiazepine recognition site of the GABA_A_ receptor complex to inhibit GABA-stimulated chloride conductance (inverse agonist effect) [155,156] (Figure 4). By using the whole cell patch-clamp technique, it was observed that LEV reversed the inhibitory effect of the methyl-6,7-dimethoxy-4-ethyl-β-carboline-3-carboxylate (DMCM, inverse agonist), *N*-methyl-beta-carboline-3-carboxamide (FG7142, partial Inverse agonist), and butyl 9*H*-pyrido[3,4-b]indole-3-carboxylate (β-CCB, inverse agonist) on GABA-elicited currents in hippocampal and cerebellar granule neurons [145]. In addition, LEV completely abolished the inhibitory effects of DMCM and β-CCB on glycine currents of spinal neurons [145]. The in vitro interaction of LEV with negative allosteric modulators of inhibitory receptors was confirmed in vivo in sound-susceptible mice; the administration of LEV (17 mg/kg) produced an important suppression of convulsions in these mice. The protective LEV effect was significantly diminished by the co-administration of FG 7142, from a dose of 5 mg/kg [145]. In addition, in postnatal day 10 rat pups, treatment with LEV decreased the severity of DMCM-evoked seizures in a dose-dependent manner when administered in doses of 10 mg/kg and greater [157].

Moreover, LEV completely reversed the inhibition by zinc of GABA and glycine evoked currents, in hippocampal and spinal cord neurons [145]. By recording functional synaptic-boutons, it was observed that the activation of GABA_A_ receptors by muscimol (a selective GABA_A_ receptor agonist) induced the inhibition of evoked excitatory postsynaptic currents (eEPSCs); later, in the continued presence of muscimol, the addition of Zn^2+^ increased the eEPSC amplitude (Zn^2+^ had no effect by itself on the eEPSC). However, when LEV was applied in the continuous presence of muscimol and Zn^2+^, there was a decrease in the eEPSC amplitude (also, LEV had no effect by itself on eEPSC, and in the presence of muscimol without Zn^2+^). Then, LEV reversed the Zn^2+^ induced suppression of GABA_A_ receptors, resulting in a decrease in glutamatergic excitatory transmission [158]. These results suggest that, also, the antagonism of allosteric Zn^2+^ modulation by LEV may be one of its mechanisms of action.

### 7.4. SV2A and GABAergic System

Despite of the fact that the SV2A protein is localized in all synaptic vesicles regardless of neurotransmitter content and its expression is similar on both glutamatergic and GABAergic terminals [159,160,161,162], a strong relationship between SV2A and the GABAergic system has been observed. In a microdialysis study on rats with a mutation of the *SV2A* gene (SV2A^L174Q^), a decrease in depolarization-evoked GABA release in hippocampus and amygdala was shown, without modification of the levels of glutamate [163,164]. In addition, in a rat model of SE induced by pilocarpine, an increase in hippocampal SV2A expression associated with GABAergic but no with glutamatergic terminals was reported [165]. Moreover, recordings of cultured hippocampal pyramidal neurons (CA1 and CA3) from SV2A KO and SV2A/SV2B DKO mice showed a decreased frequency and amplitude of spontaneous inhibitory postsynaptic currents (sIPSCs) and an increase in the frequency of the spontaneous excitatory postsynaptic currents (sEPSCs) but without any change in their amplitude [112,116]. These data agree with the observations of a great co-expression between SV2A and GABAergic neurons in the amygdala and hippocampus [163,164,166,167]. Finally, systemic administration of LEV decreased the hyperalgesia, probably enhancing GABAergic neurotransmission by different pathways, but the local administration of LEV did not act on the GABA system [168,169]. The close association between SV2A and the GABAergic system could be very important for the effects of LEV, but further studies must be completed in order to clarify this issue.

### 7.5. AMPA Receptors

Regarding the effect of LEV on glutamatergic receptors, few studies have been performed; in recordings of neuronal cortical cultures by whole cell patch-clamp, the application of kainate induced inward currents in all neurons, which were mediated primarily by the activation of AMPA receptors. The incubation with LEV in this culture, decreased kainate-induced currents by 26.5% and returned to basal after LEV washout, indicating a mild modulation of AMPA receptors [170]. This was confirmed by the administration of cyclothiazide, an AMPA positive allosteric modulator in these cells, since LEV decreased the amplitude of currents induced by cyclothiazide as well as diminished the amplitude and frequency of mEPSC [170].

### 7.6. Noradrenaline, Adenosine and Serotonin Receptors

LEV has also shown direct or indirect interaction with noradrenaline (α2A- and α2C), adenosine (A1), and serotonin (5-HT1B/1D) receptors contributing to its anti-hyperalgesic effect [11]. In an intraplantar carrageenan-induced model of inflammatory pain, the antagonists CTAP (µ-opioid receptor antagonist), BRL-44408 (α2A-adrenoceptor antagonist), MK-912 (α2C-adrenoceptor antagonist), 1,-3-dypropyl-8-cyclopentylxanthine, DPCPX (adenosine A1 receptor antagonist), GR-127935 (5-HT1B/1D receptor antagonist) and bicuculline (GABA_A_ receptor antagonist) were injected (i.p. or intraplantarly) before LEV administration (systemic, 10–200 mg/kg or local, 200–1000 nmol/paw); subsequently, it was assessed if LEV had an effect by means of paw pressure test, over time (60–300 min) [168,169]. The authors reported that LEV exerted dose and time-dependent anti-hyperalgesic activity. In contrast, the administration of all antagonists decreased this effect of LEV [168,169]. These results suggest that LEV could be a promising drug for inflammatory pain in humans.

### 7.7. Intracellular pH Regulation

Variations in the normal intracellular pH influence diverse functions in neurons, glia, and interstitial space [171]. Intracellular pH regulation is grouped into acid extrusion and acid loading. Acid extrusion is mainly accomplished by Na^+^/H^+^ exchangers, Na^+^-dependent Cl^−^/HCO_3_^−^ (chloride/bicarbonate) exchangers, and Na^+^/HCO_3_^−^ (sodium/bicarbonate) co-transporters. Acid loading is mediated by Na^+^-independent Cl^−^/HCO_3_^−^ exchangers [171,172]. Several studies have suggested that the Na^+^-independent Cl^−^/HCO_3_^−^ exchanger, AE3, may modulate seizure susceptibility [171,173]. Moreover, it has been proposed that LEV mediates pH shifts and seizure-like activity via HCO_3_^−^ regulation. In human neocortical brain slices from patients with TLE pharmaco-resistant, LEV was associated with a subtle acidification, predominantly in more alkaline cells. This acidification was depended on the extracellular HCO_3_^−^ concentration [20]. Then, since acidifications induced by LEV were based upon an inhibition of the Na^+^/HCO_3_^−^ co-transporters, Na^+^-dependent HCO_3_^−^ transporters and Na^+^-dependent Cl^−^/HCO_3_^−^ exchangers, LEV may decrease the intracellular pH by weakening the transmembrane HCO_3_^−^-mediated acid extrusion [20,174]. In addition, recordings from hippocampal slices treated with 4-aminopyridine (to increase neuronal excitability), showed that administration of LEV (10–100 µM), decreased the frequency of spontaneous action potentials and bursts of CA3 neurons. Both effects were reversible upon LEV washout and by incubating LEV plus the alkalinizing agent trimethylamine [174]. Then, by inducing intracellular acidification, LEV may attenuate the excitatory neuronal activity, promoting the termination of epileptic activity and contributing to its anticonvulsive potency [20].

### 7.8. Single or Integrated LEV Molecular Mechanism of Action?

Traditionally, the SV2A protein has been considered as the main therapeutic target of LEV. Löscher et al. proposed that although some cellular and molecular effects of LEV may contribute to its unique pharmacological profile, they have a modest magnitude [8]. However, Cortes et al. pointed out that the mechanism of action of LEV comprises a cascade of effects that in the first instance, are exerted by binding to the SV2A protein, but its pharmacodynamics involve various molecular targets that must be integrated into a single mechanism of action by a single pathway [11]. We agree with this last point of view and further propose that LEV is a unique antiepileptic agent that has multiple mechanisms of action that from an integrated point of view may explain not only its molecular effects but also their genetic, antiepileptic, antiepileptogenic, neuroprotective, antioxidant and anti-inflammatory actions (see below). Figure 4 represents the hypothetical integrated molecular mechanisms of action of LEV.

## 8. Genetic Mechanism

The antiepileptic activity of LEV has been related, besides the SV2A expression, to modifications in the expression of diverse genes. In amygdala-kindled rats, this process was associated with an upregulation of hippocampal brain-derived neurotrophic factor (BDNF) and neuropeptide Y (NPY) mRNA levels. Treatment for 12 days with LEV clearly delayed the progression of kindling, showing a clear antiseizure effect and prevented the increase in BDNF and NPY mRNA [175,176]. In addition, using real-time quantitative polymerase chain reaction temporal lobe expression of NPY gene and other epilepsy-related genes, such as, thyrotropin-releasing hormone (TRH) and glial fibrillary acidic protein (GFAP) were confirmed to be up-regulated in amygdala-kindled rats and partially normalized by LEV treatment [175]. In another work using amygdala-kindled rats, LEV 1 h prior to the kindling stimulation attenuated the hippocampal overexpression of TNF-α and Cox-2, two genes related to inflammatory processes. The decrease in the expression of both genes was parallel to the antiseizure effect of the drug [177]. LEV every day in 1 week also reduced the expression levels of interleukin-1β (IL-1β) and interleukin-1 receptor subtype I, and the associated reactive gliosis in the hippocampus and piriform cortex of epileptic rats [178].

In relation of the effect of LEV with other genetic pathologic mechanisms, Rassu et al. showed that LEV treatment ameliorated the effect of pathological mutant phenotype of leucine-rich repeat kinase 2 (LRRK2), an enzyme that controls the vesicle trafficking [179]. In this study, LEV treatment significantly decreased the neurite shortening phenotype of mutant mice in primary neurons and in PC12 cells. LEV also diminishes the accumulation of dopamine receptor D2 (DRD2) into the Golgi areas due to the mutant expression in SH-SY5Y cells. These results indicated that LEV reverts LRRK2 G2019S-associated pathological effects and that LRRK2 and SV2A are involved in a common protein network controlled by LEV with the consequent modulation of traffic and dynamics in neurons [179]. In another study, it was showed that LEV once daily for 5 days reduced the effect of presynaptic gene Stxbp1 mutations in *Stxbp1*^+/−^ mice, significantly reducing the number of spike-wave discharges. The de novo heterozygous mutations in *STXBP1*/Munc18-1 gene were implicated in the development of early infantile epileptic encephalopathies [180]. In a clinic case, LEV administered continuously from one month of age to 26 months of age had a dramatic efficacy in the treatment of encephalopathy refractory in a child with de novo heterozygous mutation (c.[922A>T]p.[Lys308(∗)]) in the STXBP1 gene [181]. Moreover, it was shown that the administration of LEV (mean duration of 50.59 ± 37.93 months) in women with a diagnosis of epilepsy caused high serum levels of the Wnt antagonists sclerostin and DKK-1 in comparison with the healthy controls. This study showed that the LEV effect is implicated the modulation of the Wnt signaling pathway [182]. In addition, it has been shown that LEV (18 and 180 μM) significantly decreases the gene expression of excitatory amino acid transporter 2, (EAAT2) in brain metastasis glioblastoma cells culture, indicating that LEV has a mechanism of action that decreases the recapture of glutamate from the extracellular space in brain cancer [183]. In addition, in a mouse microglial BV-2 cell line culture, it was shown that lipopolysaccharide increases activator protein-1 (AP-1), FOS like 1, AP-1 transcription factor subunit (FosL1), MAF BZIP transcription factor F (MAFF) and Spi-C transcription factor (SPIC) mRNA levels and LEV attenuated AP-1 and FosL1 mRNA expression in this model. Therefore, the authors suggested that LEV can be a candidate for the treatment of neurological diseases that involve microglial activation [184].

Neuronal activity influences gene expression and the drugs that modify it, through interference in neurotransmission. Therefore, LEV can affect gene expression [122,185,186]. There is evidence that in HeLa cells, the main metabolite of LEV blocks histone deacetylases which catalyze the hydrolysis of acetyl groups from the lysine of some proteins, such as histone tails, inducing chromatin condensation and inhibiting gene transcription [187]. In addition, it was observed in electrocorticography registers of epileptic patients an epileptiform electrical activity in the form of spikes, which has been associated with changes in gene expression [188,189]. Thus, if LEV can inhibit hypersynchronous neuronal activity by reducing the epileptiform activity-induced population spikes in CA3 [148] and in dentate gyrus (DG) [190], this suggests that LEV could modulate the expression of genes, as we mentioned above.

### Effect of Gene Polymorphisms in LEV Treatment in Clinical Studies

Several investigators have studied the association of some gene polymorphisms with LEV treatment (with a target dose of 20–60 mg·kg^−1^ daily for 3–4 weeks) in epileptic population. Zhao et al. studied the impact of adenosine-triphosphate (ATP)-binding cassette sub-family B member 1 (ABCB1) polymorphisms rs1128503 (C1236T), rs2032582 (G2677T) and rs1045642 (C3425T) in exon 26 on LEV serum levels and treatment efficacy in 245 Uygur Chinese children with epilepsy (drug resistant and drug-responsive). The authors showed that for genotype frequencies of ABCB1 G2677T/A, the GT genotype frequency was significantly different between drug-resistant and drug-responsive groups (OR = 0.484, 95%CI = 0.236–0.003, *p* = 0.046). The other genotype frequencies did not significantly differ between both groups [191]. In relation to the serum drug concentration of LEV and the serum drug concentration/body mass dose ratios (CDR), it was observed that rs2032582 and rs1045642 polymorphisms are significantly related to LEV concentration and CDR values. A higher LEV concentration and CDR values were found in GT, TT, GA, and AT genotype carriers of rs2032582 polymorphism in comparison with GG carriers (*p* = 0.021). Higher LEV concentrations and CDR values were found in TT genotype carriers of rs1045642 compared with CC and CT carriers (*p* = 0.002). The authors concluded that ABCB1 rs2032582 and rs1045642 polymorphisms may affect the therapeutic efficacy of LEV in epilepsy [191].

In relation to SV2A mutations, surprisingly, in a pediatric epileptic patient with a rare de novo heterozygous variant in SV2A (NM_014849.5:c.1978G>A:p.Gly660Arg), seizures were found to worsen after treatment with increasing doses of LEV for approximately 40 days [192]. Moreover, Wolking et al. investigated the genetic risk of rare variants for drug response to three AEDs (including LEV treatment for at least 1 year) through an analysis of the sequencing, genotyping, variant selection, and annotation of an epileptic cohort derived from the EpiPGX Consortium, to identify genetic biomarkers of epilepsy treatment response and adverse drug reactions [193]. The individuals who met the inclusion criteria were 1622 patients with a diagnosis of focal epilepsy and genetic generalized epilepsy. The results of the gene set analysis showed a significant enrichment of protein truncating variants and splice-region variants in the SV2 gene group (SV2A and SV2B) associated with drug resistance. Thereafter, it was possible to conclude that a group of genes are related to drug kinetics or targeting in drug resistance to LEV [193].

In another study, through the screening for mRNA signatures in 53 epileptic hippocampal tissue from pharmacoresistant TLE patients, abundant synapse-associated molecule mRNA signatures in LEV a priori non-responders were shown. In the promoter characterization was observed an accumulation of the rs9305614 G-allele in phosphatidylinositol *N*-acetylglucosaminyltransferase promoter with activation of LBP-1 transcription factor of LEV a priori non-responders mesial TLE patients. The authors suggest that epigenetic factors predisposing for a priori LEV pharmacoresistance by transcriptional targets [194]. Finally, in another study, the presence of rs1611115, rs4680 and rs1800497 polymorphisms with adverse psychotropic side effects of LEV in patients with chronic epilepsy was found to be related with a decrease in dopaminergic activity [195].

## 9. Anti-Ictogenic Mechanism

LEV differs from classical AEDs since it does not share affinity with targets of other AEDs such as valproic acid, phenobarbital or benzodiazepines and does not work by the three classic routes of AEDs: sodium channel modulation, low-voltage-activated (T-type) calcium channel modulation and a direct GABAfacilitation [99,196]. Furthermore, LEV was devoid of anticonvulsant effect in the maximal electroshock seizure (MES) and pentylenetetrazol (PTZ) seizure tests [197,198], which are traditionally used to evaluate the effectiveness of AEDs [199], but it markedly suppresses seizures in chronic models such as mice kindled with corneal electroshocks, genetically epileptic animals, and epilepsy models induced by pilocarpine or kainic acid, providing evidence for its anticonvulsant properties [198,200]. The action of LEV to protect selectively against seizures in chronic models distinguishes it from other AEDs that act in both normal and “epileptic” animals [200]. This is consistent with intracellular slice recordings from CA3 neurons, where LEV does not influence normal glutamatergic and GABAergic transmission but instead decreases the epileptiform activity induced by the GABA_A_ receptor antagonist bicuculline or the glutamate agonist *N*-methyl-D-aspartate (NMDA) [201]. This selective activity appears to be related with a different mechanism of action which includes only inhibition of burst firing without interference with normal neuronal excitability, resulting in protection against the transition from interictal to ictal activity. On this basis, it is suggested that LEV preferably act on neurons that exhibit abnormal patterns of activity. This probably explains the lack of anticonvulsant activity against acute seizures [198] compared with their potent anticonvulsant action in chronic models, as well as its unique tolerability and safety [202].

There are multiple preclinical and clinical studies that showed the anticonvulsant activity of LEV. In the pilocarpine model of TLE the administration of the drug by osmotic minipumps showed a reduction in the frequency of spontaneous recurrent seizures (SRS) during one [150] and two weeks of treatment [203]. Interestingly, the individual responses of the rats during two weeks of treatment varied markedly from full seizure control to no effect [203], likewise occurring in epileptic patients [194]. The results of the EEG from spontaneous epileptic rats (SERs), showed that both a single (80 or 160 mg/kg, i.p.) or repeatedly (80 mg/kg/day, i.p. for 5 days) administration of LEV decreased the number of tonic convulsions and absence-like seizures. Furthermore, long-lasting seizure protection by LEV after cessation of treatment was observed [204]. The pretreatment with LEV (30–200 mg/kg, i.p.) prevented the development of the SE induced by pilocarpine and decreased neuronal death in mice [205]. In amygdala-kindled rats, LEV decreased the seizure intensity and duration after initial dosing; however, chronic treatment (three times daily i.p., at a dose of 108 mg/kg for 21 days) decreased the anticonvulsant efficacy indicating a development of tolerance [206]. In a novel model of extended hippocampal kindling in mice the administration of LEV at 400 mg/kg significantly decreased the number of SRS at 10–12 h post-injection [207]. Additionally, to the anti-ictogenic effect in animal models of TLE, LEV administration (8 and 16 mg/kg/h; 7-day period via an osmotic minipump) reduce the frequency of focal and generalized-onset seizures in the tetanus toxin model of focal impaired awareness epilepsy (before known as partial complex seizure) [208]. LEV was also effective in animal models of chronic epilepsy, including genetic models (Genetic Absence Epilepsy Rat from Strasbourg, GAERS) and WAG/Rij rats [209,210], as well as in a rodent model of neonatal hypoxic seizures, where LEV pretreatment (50 mg/kg) resulted in a decrease in both behavioral seizure duration and in the duration of ictal electro encephalographic activity during hypoxia [211]. In chronic epileptic rats treated repeatedly with LEV (564 mg/kg/day 1 week via osmotic minipumps) show less severe seizure behavior during drug treatment [212]. Similarly, intravenous LEV treatment 30 min after the onset of pilocarpine-induced acute seizures decreased the intensity of the seizures [213]. The evidence displayed above shows that LEV reduces the number and intensity of the seizures. The drug offers broad-spectrum seizure protective activity in a variety of animal models, including models, generalized, and focal-onset seizures.

There are already 132 clinical trials about the effect of LEV in patients with epilepsy, of which 90 have been completed [214]. Some of them are explained in detail in a previous section (clinical indications). The results are in line with the preclinical observations and indicate that add-on LEV treatment can reduce the seizure frequency in epileptic patients.

Several electrophysiological analyses have shown that LEV can reduce some epileptic traces. Levesqué et al. found a reduction in interictal spikes rates in CA3, entorhinal cortex, DG and subiculum in LEV-treated rats (300 mg/kg/day for 2 weeks) without seizures [21], similar observations were performed in patients with focal-onset seizures and acute (500 mg twice daily) or chronic treatment with LEV (individualized, 500–1000 mg twice daily, over 8 weeks) [215]. Furthermore, the analysis of interictal high-frequency oscillations show that LEV can decrease both ripples and fast ripples (predictors of seizure occurrence) particularly in CA3, which suggest that antiseizure LEV activity could be related with inhibit excessive synchronization in CA3 [21], as well as between hippocampus and cortex [148].

**Figure 5:**
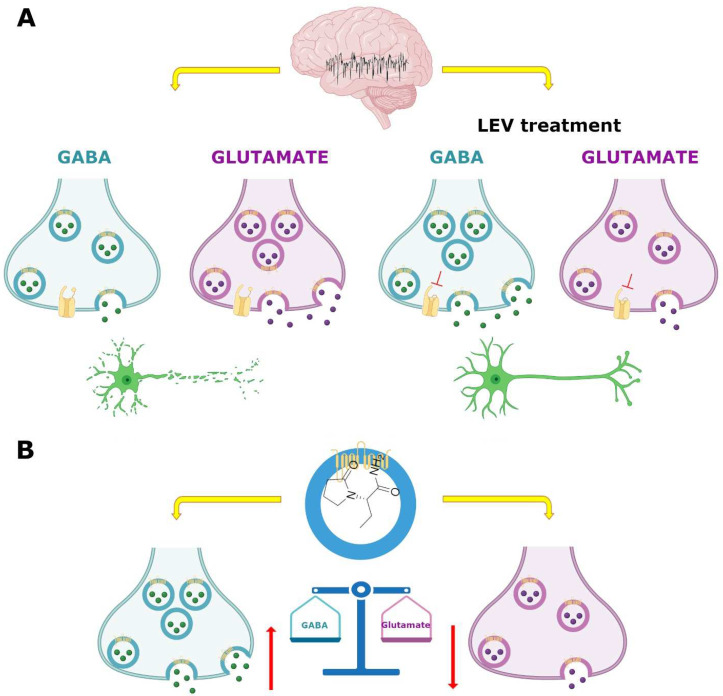
Hypothetical acti-ictogenic and neuroprotector effect of levetiracetam (LEV). (**A**) The anti-ictogenic effect of LEV seems to be an integrative mechanism, that involved the interaction to this AED with different pharmacological targets, resulting in a decrease in the excitability in the epileptic circuit. Although the exact mechanism throughout LEV can decrease the excitability remain unclear, the evidence suggests that SV2A plays a critical role. In epileptic nerve terminal, the binding LEV-SV2A could result in augment or decrease in neurotransmitter release. Moreover, LEV blocks the voltage calcium and decreases the release of the neurotransmitters. (**B**) GABA and glutamate are the principal neurotransmitters responsible for maintaining a balance between inhibition and excitation, respectively. In epilepsy, as well as in other pathologies that cause neuronal damage, there is an imbalance between these systems, which results in increased excitability and neuronal death. LEV decreases both events, perhaps by modulating neurotransmitter release by binding to SV2A, which may result in increased neurotransmitter release, particularly GABA, or in a decreased of neurotransmitter release, particularly glutamate. Regardless, whether LEV particularly increases GABA release or decreases glutamate release, the final effect is decreased excitability, seizures, and neuronal death by restored the balance between excitatory and inhibitory systems.

The anti-ictogenic effect of LEV seems to be an integrative mechanism that involves the interaction of this AED with different pharmacological targets (as mentioned before), notably by its action on SV2A, resulting in a decrease in the excitability in the epileptic circuit. However, the exact mechanism by which LEV decreases the excitability still remains unclear. The microdialysis analysis in epileptic rats has shown that LEV (300 mg/kg/day for one week) facilitates the vesicular release of neurotransmitters, with a preferential effect on GABA [150]. In contrast, LEV decreased excitatory and inhibitory synaptic currents in an in vitro study of whole cell patch recordings, which suggest that LEV binds to SV2A and reduces presynaptic neurotransmitter release [122,216]. This is in line with the data about the blockade of presynaptic calcium channels and the decrease in glutamate decrease preferentially [217]. Regardless, whether LEV particularly increases GABA release or decreases glutamate release, the final effect is decreased excitability and seizures. Finally, although LEV can act on all those terminals where SV2A is expressed, apparently their effects could be finely modulated by neuronal activity, pathophysiology of the nervous tissue and by SV2A expression [111]. In Figure 5, we propose based on the experimental data that the possible LEV anti-ictogenic effect is mainly due to the interaction with SV2A, but as mentioned above, LEV could interact with other pharmacological targets to reduce excitability and seizures.

## 10. Antiepileptogenic Mechanism

The potential antiepileptogenic and disease-modifying effect of LEV, are evidenced in kindling and genetic epilepsy models. LEV could interfere with the modification of the circuits involved in seizure development, since it delays the deleterious effects of kindling [218,219]. However, in SE models, the results are controversial; for example, rats with chronic LEV treatment for 3 weeks in different doses (50, 150 and 300 mg/kg/day osmotic via), after SE, showed a decrease in hippocampal hyperexcitability, reducing the population spike amplitude in the DG, but it did not prevent the development of spontaneous seizures [190]. Other studies have explored this issue. Table 1 summarizes the principal results in the pre-clinical trials.

Moreover, clinical studies about the antiepileptogenic effect of LEV have been conducted in patients with intracerebral hemorrhage, traumatic brain injury (TBI), supratentorial neurosurgery and spontaneous subarachnoid hemorrhage which have a higher epilepsy risk [226]. Although the current data is heterogeneous, contradictory and neither supports nor refutes the use of LEV for seizures prophylaxis [227,228,229], LEV treatment has shown interesting results; therefore, an evaluation of its antiepileptogenic potential is worthwhile.

In a retrospective study of patients followed for one year receiving LEV (doses from 500 mg to 3000 mg a day; most frequent dose being 1000 mg a day) or phenytoin (doses from 200 to 800 mg a day, being the most frequent 300 mg) after supratentorial craniotomy, LEV treatment demonstrated a non-significant reduction in seizure incidence after 1 year and fewer early adverse reactions than phenytoin [230]. A prospective, randomized study with prophylactic LEV treatment (500 mg/body every 12 h until postoperative day 7) in the perioperative period, showed a reduction in seizure incidence [231]. In patients with TBI, LEV was used at 55 mg/kg daily for 30 days, starting on day 8 after injury. After two years, 5 of 46 treated adults (10.9%) and 8 of 40 untreated adults (20.0%) developed post-traumatic epilepsy (relative risk, 0.47; *p* = 0.18). Even though, LEV treatment non-significantly reduced posttraumatic epilepsy cases, it was safe and well tolerated [232]. In contrast, Kruer et al. showed a decrease in seizure incidence seven days after TBI in patients treated with phenytoin and LEV [233]. Radic et al. reported that LEV (1000 mg intravenous loading dose, followed by a dose of 500–1000 mg intravenous or orally twice daily) prevented clinical and/or electrographic seizures after acute/subacute subdural hematoma diagnosis [234]. In another clinical study with newborns, LEV maintained for one month after the seizures resolved) counteracted the detrimental effect of seizures on the neurodevelopmental outcome, specifically tone and posture [235]. These findings support the further evaluation of LEV treatment as antiepileptogenic therapy in patients after TBI.

Furthermore, intravenous LEV for 3 days and oral regimen at 90 days has been proposed as an optional drug for the prevention of seizures and epilepsy in craniotomy and other neurosurgical interventions [236]. In a study of intracerebral hemorrhage patients, the prophylactic use of LEV (delivered at a rate of 500–2000 mg/d) improved the prognosis and was more effective than phenytoin (15–20 mg/kg) in preventing seizures, without affecting cognitive ability [237]. Moreover, a recent study indicated that epileptic patients treated with LEV showed increased functional connectivity compared to healthy subjects [238], relating to normalization of left medial temporal lobe deactivation during verbal performance [239].

**Figure 6:**
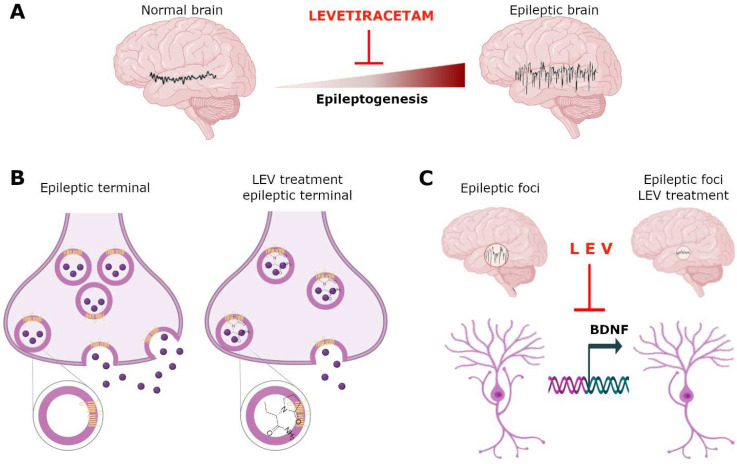
Putative antiepiletogenic levetiracetam (LEV) mechanism. (**A**) LEV is one of the few antiepileptic drugs (AED) able to retard or inhibit the generation of epileptic neural circuits. The mechanism through which it does is not completely elucidated, but apparently inhibiting the excessive synaptic transmission. (**B**) The binding LEV-SV2A improve the SV2A effects, diminishing the hyperexcitability and thus delays epileptogenesis. (**C**) LEV inhibit epileptic foci formation by suppressing BDNF synthesis and consequently the mossy fiber sprouting.

The mechanisms involved in the antiepileptogenic effect of LEV are not fully understood. However, several mechanisms could explain its antiepileptogenic or disease-modifying activity. LEV may inhibit epileptogenesis through an increase in the suppressive effect of SV2A: (1) DKO mice (SV2A and SV2B) showed an increase in synaptic transmission during repetitive presynaptic fiber excitation [110] (2) in amygdala and corneal kindling epilepsy models, SV2A (+/−) mice show accelerated epileptogenesis [128]. This suggests that SV2A function inhibits or modulates excessive synaptic transmission during kindling. Then, LEV binding to SV2A may improve the SV2A inhibitory effects on hyperexcitability. Further support for an LEV antiepileptogenic effect derives from observations showing that at chronic treatment (21–28 days) and high-dose (150–500 mg/kg) attenuates hippocampal cell death and hippocampal excitability following pilocarpine-induced SE [22,190]. Another possible LEV mechanism is to inhibit epileptic foci formation, since it suppresses seizure-induced increase in neurogenesis in the kainate-induced SE model after 25 days of treatment (320 µmol/L released osmotic pathway to 0.21 µL/h) [224] and their aberrant migration from the dentate subgranular zone to the hilus after 21 days of treatment (100 mg/kg, i.p.) [222]. This effect appears to be mediated by suppressing BDNF synthesis induced by kindling in chronic treatment of 12 days (100 mg/kg/day; once daily, 5 days per week) [175,176] and related to mossy fiber sprouting and consequently neuronal excitability [240] (Figure 6).

## 11. Neuroprotective Mechanism

In addition to the indirect neuroprotective effect of LEV, through its anti-ictogenic and anti-epileptogenic mechanisms mentioned above, there are several studies that show a direct neuroprotective effect in different pathological conditions where the brain has suffered an injury, for example, in brain ischemia, which has been also associated with post-traumatic epilepsy [187,241].

Previous administration of LEV (50 mg/kg) in rats injured with kainic acid resulted in a significant decrease in the death of hippocampal CA1 neurons and this was partially mediated by inhibition of lipoperoxidation [242]. Analysis with the neurodegeneration marker Fluoro-Jade B showed that the administration of LEV 2 h after the onset of SE at 100 or 150 mg/kg was effective in protecting against neuronal damage induced by this condition, in CA1 and CA3 pyramidal cell layers as well as the dentate hilus compared to diazepam and valproate. In contrast, when LEV was administered as an adjunct to diazepam, the neuroprotective effect was not observed compared to when it was administered alone [243]. Histological examination with cresyl violet staining showed that a high dose of LEV (500 mg/kg for 28 days) significantly reduced the number of neuronal cells lost in the CA1 and CA3 and DG areas at two days after SE and it protected against the sequence of excitotoxic events induced by vasogenic edema [22]. A year later, they found through MRI studies in mice, that 3 h after SE there was cytotoxic edema without injury in the BBB, whereas by 2 days post-SE, vasogenic edema was developed in the dorsal hippocampus, amygdala and piriform cortex, which related with the loss of BBB integrity, the latter was markedly ameliorated by LEV treatment (30 min after diazepam injection, and thereafter twice a day for 7–10 days; 350 mg/kg), as well as reactive astrogliosis. These results suggested that the prevention of the development of epilepsy by LEV treatment was associated with the protection of the BBB via astrocytes and the inhibition of angiogenesis in the hippocampus; since the drug treatment significantly reduced the mRNA expression of some angiogenic factors (angiopoietin-2, Tie-2, vascular endothelial growth factor-A VEGF and its receptor) increased by SE [244,245]. Moreover, some reports have shown that LEV did not prevent acquired epilepsy when administered in post-SE animal models of TLE, although it did exert neuroprotective effects [225,246,247].

The neuroprotective effect of LEV has also been evaluated in models of focal cerebral ischemia, where it was found to be highly effective in reducing the infarct volume without altered body temperature, with better results than MK-801 (dizocilpine, non-competitive *N*-methyl-D-aspartic acid antagonist) [248]. Likewise, in neonatal rat model hypoxic–ischemic brain injury, seven days of LEV treatment (40 mg/kg/day) significantly decreased the numbers of TUNEL positive apoptotic cells in both the hippocampus and cerebral cortex [249]. Additionally, in a recent study it was found that LEV treatment (150 mg/kg for 14 days) promoted angiogenesis and functional recovery in a model of cerebral ischemia in rats. These effects appear to be mediated by anti-inflammatory and anti-apoptotic activities, in addition to inducing the expression of heat shock protein 70 (HSP70), VEGF, and hypoxia inducible factor 1α (HIF-1α) [250]. In models of head trauma and hemorrhage in the subarachnoid space, the use of LEV in mice at a dose of 54 mg/kg for 3 days showed an improvement in vestibular and motor functions, in addition to a reduction in neuronal histological damage [251]. The administration of LEV (50 mg/kg) in rats with intracerebral hemorrhage improved the neurological function 24 h after injury; moreover, it decreased the brain water content, the expression of NF-kB and the damage to neurons around the hematoma [252].

LEV (25 mg/kg; for six weeks) also showed neuroprotective effects in the neuropathy derived from diabetes; in mice, the regulation of the expression of apoptotic markers Tp53 and iNOS (tumor protein 53 and inducible nitric oxide synthase, respectively), as well as some neuronal indicators of stress and microglial activation, such as GFAP was decreased. Moreover, LEV increased the regulation of the neuronal marker of regeneration growth associated protein 43 (GAP43) [253]. Finally, the neuroprotective effect of LEV was evaluated in a model of spinal cord injury; the acute treatment with this drug resulted in an improvement in gross and fine motor functions. Histological analysis demonstrated that LEV treatment significantly reduced lesion size and protected the motor neurons of the corticospinal tract. On cervical injuries, a neuroprotective effect was observed by LEV (4 h post injury) on neuronal fibers and survival of oligodendrocytes [254].

The fact that LEV decreases the damage in different models characterized by neuronal injury (ischemia, TBI and spinal cord injuries), suggests that this condition shares some pathophysiological events with epilepsy [187]. The possible mechanisms of the neuroprotective effect of LEV in neuronal injury are not completely clear, but it may be related to the ability of LEV to modulate the release of neurotransmitters, particularly GABA and glutamate, since one of the events that initiated neuronal damage is an increase in glutamate and a decrease in GABA release, producing an imbalance between excitation and inhibition [255]. The release of glutamate and their interaction with the postsynaptic receptor NMDA augment the influx of Ca^2+^ into cells, triggering the cascade of events that underlies cell death, so, it is possible that selective blockade of calcium channels may be neuroprotective. In this sense, LEV could be considered, because it regulates the influx of calcium into the cells [109] and blocks N, P/Q and L subtypes calcium channels [136,137,138,139]. Moreover, LEV acts through the ryanodine and IP3 receptors inhibiting Ca^2+^ release [142,143]. Thus, LEV could prevent the progressive deterioration of epilepsy and protect the brain against cytotoxicity and neuronal death [256]. Moreover, GABAergic neurotransmission is decreased during brain ischemia and epilepsy [257], therefore strategies that enhanced it, may be result in decrease in cellular damage. We show through a microdyalisis analysis that LEV treatment for one week (300 mg/kg/day) can induce an increase to GABA release in response to potassium-induced depolarization in the hippocampus of epileptic rats and restored the GABA/glutamate balance [150]. Further studies must be completed to better understand this phenomenon.

## 12. Anti-Inflammatory and Antioxidant Mechanisms

Epilepsy triggers neurodegenerative and inflammatory mechanisms that occur parallel to epileptic seizures. Emerging studies have shown that the production of excessive ROS causes an alteration in biochemical and molecular brain processes, triggering neuroinflammation. Both processes (ROS and inflammation) promote neuronal hyperexcitability, seizures and are crucial factors in the onset and progression of neurodegeneration in TLE [258,259,260], as well as the subsequent synaptic reorganization and cognitive deterioration in epileptic patients [259,261,262,263,264,265,266].

Treatments aimed at reducing inflammation and ROS in epilepsy, in addition to controlling epileptic seizures, have resulted in better management of the epileptic patient. For example, the use of antioxidants can prevent the epileptic process in animal seizure models [267] and in a recent work, we showed that valproic acid, administered by 12 months without interruption acts as an antioxidant in children diagnosed with epilepsy decreasing oxidative markers as malondialdehyde (MDA, a lipid peroxidation marker), 8-hydroxy-2-deoxyguanosine (8-OHdG, DNA oxidation marker), 3-nitrotyrosine (3-NT, protein oxidation marker), and hydrogen peroxide (H_2_O_2_) levels and increasing glutathione peroxidase (GPx) levels, catalase (CAT), glutathione reductase (GR) and superoxide dismutase (SOD) antioxidant enzyme activities [268]. However, the antioxidant effect of AEDs is still a matter of debate [269].

Recent evidence in different animal models, suggests that LEV exerts neuroprotective effects via anti-inflammatory and antioxidant effects [178,244,270]. In the liver of male Wistar rats, the reduced glutathione (GSH) level significantly increased after an acute dose of LEV prior induction to *SE* (50 mg/kg i.p.) and in LEV plus carbamazepine [271]. In the TBI model, LEV (50 mg/kg i.p.; once a day for 20 days) administration significantly inhibited the thiobarbituric acid reactive substances (TBARS) levels (a lipid oxidation marker). Moreover, SOD and GSH levels were also significantly increased [272]. The same observation was shown in the kindling model of epilepsy-induced by PTZ, where the nitrite, 8-OHdG, and lipoperoxidation levels were significantly reduced, and GSH, GPx, and SOD were increased for chronic treatment with LEV [273,274]. The last evidence shows that with high doses of LEV (300 mg/kg i.p.) in a kindling model of epilepsy induced by PTZ in rats, the oxidant status in epilepsy was decreased with the use of this AED [24,275]. In the SE model induced by pilocarpine was shown that pretreated with LEV (200 mg/kg i.p.) decreased high levels of 8-isoprostanes (a marker of oxidative stress), nitrite-nitrate levels, CAT, and GSH (*p* < 0.05) in the hippocampus [276,277]. Likewise, the neuroprotective effect of LEV (54 mg/kg, i.p. administered for 7 days) through its antioxidant action has also been observed in the striatum of rats that were administered a neurotoxin that induces excitotoxicity [278]. In a rat model of Parkinson’s disease induced by rotenone it was shown that LEV (600 mg/kg, i.p. for 21 days) significantly decreased the lipoperoxidation levels and increases GSH, CAT, and SOD levels in the brain [279]. In addition, in a rat model of diabetic neuropathy induced by streptozotocin was shown that the treatment for one month with LEV (300 and 600 mg/kg, i.p.) reduces lipoperoxidation, and increases total antioxidant capacity [280,281]. However, a toxicity of LEV (310 mg/kg, v.o.) due to a significant increase in MDA levels and a decrease in GSH and SOD levels (*p* < 0.05) was observed in the homogenized brain tissue of rats after the administration for 45 days of this drug [282]. Moreover, the reproductive toxicity of LEV (150 and 300 mg/kg, v.o.), has been reported where this AED induces DNA damage in the rat testes and decrease GSH, SOD, and CAT levels after 70 days of treatment [283].

Several clinical studies have shown that LEV modified the antioxidant/oxidant status in patients with epilepsy [284,285], this AED increased the oxidative state (MDA, 15F-2t-isoprostane and 8-OHdG markers) in patients diagnosed with epilepsy [286,287], and significantly increase the 8-OHdG levels in combination with valproate [288]. On the other hand, this AED improved the antioxidant status in the patients with intellectual disabilities and in patients diagnosed with epilepsy; determined through of reactive metabolite oxygen levels, oxidized low-density lipoprotein, paraoxonase, and arylesterase activities [288,289,290,291]. These data indicate the need more clinical trials for obtaining reliable results of the antioxidant properties of LEV.

Regarding neuroinflammation, treatment with LEV (360 mg/kg; for 7–10 days) suppressed the expression of proinflammatory molecules, such as tumor necrosis factor α (TNF- α), interleukin 6 (IL-6), interleukin 1β (IL-1β), and iNOS, 1 or 3 h after SE as well as prevented the increase in the numbers of Iba1-positive microglia and the conversion to an amoeboid-like shape from a ramified shape after SE [244]. During epileptogenesis, repeated treatment with LEV for 30 days prevented microglial activation, as evidenced by a decrease in morphological changes, phagocytic activity, and cytokine expression [23]. Additionally, in epileptic rats, LEV (30 mg/kg, i.p. for 1 week) reduced reactive gliosis and the expression levels of IL-1β and interleukin 1 receptor type I in the hippocampus and piriform cortex [178]. Finally, the anti-inflammatory action of LEV has also been demonstrated using in vitro assays in microglial/astrocyte cocultures [292].

In addition, LEV treatment restored impaired astroglial gap junction coupling, along with repolarization of the membrane rest potential, under inflammatory conditions [293], and it increased the expression of growth factor beta 1 (GF-β1) [292]. The anti-inflammatory effects of LEV also were showed in diabetic mice; treatment with LEV (25 mg/kg) for six weeks reduced the transcription of proinflammatory molecules as NF-κB, TNF-α, IL-6, iNOS and Tumor protein 53 (Tp53), as well as regression of retinal inflammation and improvement in structural organization [253]. Using the BV-2 cell line and liposaccharide stimulation (inflammatory model of microglia in vitro), it was demonstrated that 50 μg/mL of LEV decreased the mRNA levels of IL-1β and TNF-α, and significantly decreased protein expression of NF-kB-light-chain-enhancer of activated B cells and signal transducer and activator of transcription 3 (STAT3; [252]). Moreover, in a mouse model of multiple sclerosis has been showed that LEV modulates the gene expression of IL-1β and tumoral growth factor-1beta (TGF-1β) acting as an anti-inflammatory [294]. Hansson et al. observed that, by combining LEV, glucose (high concentration), naloxone (low concentration), sildenafil, endorphin-1, and vitamin D3, Ca^2+^ signaling was restored in reactive astrocytes and, thus, they returned to their normal physiological state restoring its vital functions [270]. Taken together, the observations suggest that the efficacy of LEV derives, in part, from its ability to prevent astroglial inflammatory activity and the production of pro-inflammatory molecules via the inhibition of microglia activation and signaling pathways. In contrast, in serum samples from rats with spinal cord injury, LEV did not decrease levels of IL-1β, IL-6, IL-10, interferon γ (IFNγ), TNFα, and IL-4 in 1- and 24-h post injury [254].

The clinical evidence regarding the anti-inflammatory effect of LEV is limited. The prospective clinical trial of 21 epileptic patients treated with LEV (1464 ± 405 mg/day), showed a decrease in the percentage of T-lymphocytes (CD4^+^ CD25^+^) without significant changes in leukocytes, neutrophils, total lymphocytes, and cytokine levels (IL-1β, IL-6, TNF-α and monocyte chemoattractant protein-**1**, MCP-1) in the peripheral blood [295]. Similarly, in the serum of children with epilepsy, LEV or valproate treatment for 16 weeks does not change the serum IL-1β levels but decrease levels of the inflammatory marker C–C motif ligand 2 (CCL2). Additionally, both drugs reduced anxiety and improved quality of life in these pediatric patients [296]. In contrast, Gulcebi et al. found a decrease in the IL1- β concentration in patients on monotherapy with LEV for at least one month [297].

**Figure 7:**
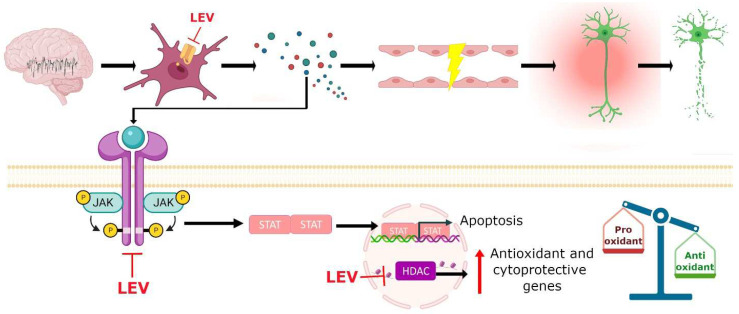
Anti-inflammatory effect of LEV. Seizures induces the activation of microglia, which in turn releases proinflammatory mediators, such as TNF-α, IL-1β, IL-6, etc. These cytokines promote the brain blood– brain barrier (BBB) damage and sustained neuronal inflammation leading to seizures and neurodegeneration, which activates further inflammation, establishing a vicious circle. The anti-inflammatory mechanism of LEV may be related to the voltage-activated Ca^2+^ channel inhibition present in the microglia (purple cell). This results in glia non-activation and thus the attenuation of inflammation. LEV also inhibits the JAK2-STAT3 signaling pathway. LEV can act as an HDAC inhibitor promoting the transcription of antioxidant and cytoprotective genes, restoring the oxidation–reduction balance.

The mechanism by which LEV exerts the anti-inflammatory and antioxidant effects has not yet been established. Before, it was proposed that LEV may act as histone deacetylase (HDAC) inhibitor suggesting that this AED shows antioxidant and anti-inflammatory properties by its probable capacity of activating master regulators of antioxidant and cytoprotective genes and antioxidant response elements [298,299] and has been demonstrated that LEV protects against mitochondrial dysfunction in an experimental model of SE [246]. The ability of LEV to decrease the inflammatory response and neuronal damage appears to be mediated by regulating the Janus kinase 2, JAK2-STAT3 signaling pathway [252]. Moreover, LEV can inhibit N- P/Q and L-type calcium channels [136,137,138,139], which results in a decrease in the intracellular Ca^2+^ concentration required for the release of cytokines and chemokines from microglia activated responsible for neuroinflammation [300], suggesting that certain Ca^2+^ channels in microglia might be potent targets of LEV (Figure 7).

## 13. Rebound Effect and Aggressiveness Behavior

Lastly, it is important to mention that LEV, similar to all drugs, has adverse effects that can limit its use. Some of them have been mentioned before (see Clinical Indications in Epilepsy and Genetic Mechanism), but there are two interesting and little explored adverse events linked to the use of LEV: the rebound effect and aggressive behavior. The rebound effect is when an abrupt withdrawal from AEDs is followed by increased occurrence of epileptic seizures. This has been observed in patients with epilepsy, which have shown an increase in the frequency of focal-onset seizures or the switch of focal to bilateral tonic-clonic seizures [301]. In a TLE model induced by kainic acid has been reported a rebound effect after rapid LEV withdrawal [302]; nevertheless, patients with refractory focal-onset seizures and withdrawal of LEV did not show this phenomenon [303]. In addition, LEV can induce psychiatric and behavioral adverse reactions, for example, depression, anxiety, psychosis, and aggressive behavior with higher prevalence in children and adolescents than in adults [304]. Aggressive behavior in epilepsy has been the subject of many misconceptions and controversies, however, it can be defined as “a social behavior that is aimed at eliciting discomfort, pain, or physical damage, to oneself, to another person, or to things or at defending oneself against a threat” [304,305]. Aggressiveness has a complex and multifactorial background in people with epilepsy [304] and LEV together with BRIV, perampanel and topiramate have been associated with higher risk of aggressive behavior than other AEDs [304,306]. Deeper studies with better-defined terms can help researchers and physicians to understand the risk of these events in order to generate profiles of effective treatment.

## 14. Conclusions

The evidence in the literature displayed in the current review indicates that LEV has molecular and biochemical mechanisms, different from its action on SVA2, that allow this drug to exert antiepileptic, antiepileptogenic, anti-inflammatory, neuroprotective and antioxidant effects. These suggest that LEV administration can address some of the current necessities in epilepsy and in other conditions and can be used in drug repositioning.

## Figures and Tables

**Table 1:** The antiepileptogenic role of LEV.

Autor	Model	LEV Treatment	Findings
Löscher et al. [218]	Rat amygdala kindling model	13, 27 or 54 mg/kg i.p. (16 days)	After daily LEV (54 mg/kg), behavioral seizure and amygdala after discharge duration remained shorter.
Stratton et al. [219]	Rat amygdala kindling model	50 mg/kg i.p. (16 days)	LEV blocked seizure development when administered 1 h prior to electrical stimulation during the kindling phase. This effect continued after washout, suggesting that LEV blocked the underlying kindling mechanism and not simply masked seizure expression by its anticonvulsant action.
Vinogradova and van Rijn [220]	Rat audiogenic kindling model	50 mg/kg i.p. (one)	A single LEV injection significantly suppressed kindling progression. This is evidence of a long-lasting antiepileptogenic activity.
Yan et al. [221]	Spontaneously epileptic rat	80 mg/kg/day i.p. (from postnatal weeks 5 to 8)	Long-term LEV treatment inhibited development of seizure activity and this effect was still evident 5 weeks after cessation of treatment.
Sugaya et al. [222]	Rat perforant path kindling model	100 mg/kg, i.p. (21 days)	LEV inhibited both the development of kindling-induced potentiation in perforant path-granule cell synapses and development of perforant path kindling.
Leo et al. [223]	WAG/Rij rats Rat model of absence epilepsy	80 mg/kg/day i.p. (17 weeks)	LEV showed antiepileptogenic effects 1 month after discontinuation. However, it did not maintain its antiepileptogenic effect 5 months after suspension, and worsened depressive-like behavior.
Itoh et al. [22]	Mice, SE induced with pilocarpine	500 mg/kg v.o. (28 days; twice a day)	LEV treatment prevented the development of spontaneous recurrent seizures for at least 28 days.
Sugaya et al. [224]	Rat kainate-induced SE model	100 mg/kg, i.p. (21 days)	LEV treatment decreased the duration of spontaneous electrographic seizures 58 days after SE.
Brandt et al. [225]	Rat, SE induced by sustained electrical stimulation of the basal amygdala	1000 mg/kg Via osmotic minipumps (5 weeks)	LEV treatment did not prevent seizure development when administrated 4 after SE onset.

## Data Availability

Not applicable.

## Abbreviations

3-Nitrotyrosine (3-NT); 8-Hydroxy-2-Deoxyguanosine (8-OHdG); Activator Protein-1 (AP-1); Adenosine-Triphosphate (ATP)-Binding Cassette Sub-Family B Member 1 (ABCB1); Alpha-Amino-3-Hydroxy-5-Methyl-4-Isoxazole Propionic Acid (AMPA); Angiopoietin-2 (Tie-2); Antiepileptic Drugs (AEDs); Blood-Brain Barrier (BBB); Bradykinin (BK); Brain-Derived Neurotrophic Factor (BDNF); Brivaracetam (BRIV); Butyl 9H-pyrido[3,4-b]indole-3-carboxylate (β-CCB); Calcium-Sensor Synaptotagmin protein (SYT-1); cAMP-Triggered Phosphorylation of cAMP Response Element Binding Protein (pCREB); Catalase (CAT); Concentration/body mass Dose Ratios (CDR); Excitatory Postsynaptic Current (EPSCs); Cytochrome P450 (CYP450); Dentate gyrus (DG); Dopamine Receptor D2 (DRD2); Double KO (DKO); Egtazic Acid (EGTA); Electroencephalogram (EEG); European Medicines Agency (EMA); Evoked Excitatory Postsynaptic Currents (eEPSCs); Excitatory Amino Acid transporter 2 (EAAT2); Excitatory Postsynaptic Potential (EPSP); FOS like 1, AP-1 Transcription Factor Subunit (FosL1); gamma-aminobutyric acid (GABA); GABA-Transaminase (GABA-T); Gamma-Aminobutyric Acid A receptor (GABA_A_); Genetic Absence Epilepsy Rat from Strasbourg (GAERS); Glial Fibrillary Acidic Protein (GFAP); Glutamic Acid Decarboxylase (GAD); Glutathione (GSH); Glutathione Peroxidase (GPx); Glutathione Reductase (GR); Growth Associated Protein 43 (GAP43); Growth Factor Beta 1 (GF-β1); Heat Shock Protein 70 (HSP70); High-Voltage Activated (HVA); Histone Deacetylase (HDAC); Hydrogen Peroxide (H_2_O_2_); Hypoxia Inducible Factor 1α (HIF-1α); interleukin 6 (IL-6); Inducible Nitric Oxide Synthase (iNOS); Inositol-3-Phosphate (IP3); Interferon γ (IFNγ); Interleukin-1β (IL-1β); International League Against Epilepsy (ILAE); Janus kinase 2 (JAK2); Knockout (KO); Leucine-Rich Repeat kinase 2 (LRRK2); LEV Binding Site (LBS); Levetiracetam (LEV); MAF BZIP Transcription Factor F (MAFF); Malondialdehyde (MDA); Maximal Electroshock Seizure (MES); Methyl-6,7-dimethoxy-4-ethyl-β-carboline-3-carboxylate (DMCM); Monocyte Chemoattractant Protein-1 (MCP-1); Multiple Sclerosis (MS); Neuropeptide Y (NPY); N-methyl-beta-carboline-3-carboxamide (FG7142); N-Methyl-D-Aspartate (NMDA); NSF Attachment Proteins (SNAPs); Nuclear Factor Kappa-Light-Chain-Enhancer of Activated B cells (NF-kB); Pentylenetetrazole (PTZ); Reactive Oxygen Species (ROS); Readily Releasable Pool (RRP); Signal Transducer and Activator of Transcription 3 (STAT3); soluble NSF attachment proteins (SNARE); Spi-C Transcription Factor (SPIC); Spontaneous Epileptic Rats (SERs); Spontaneous Excitatory Postsynaptic Currents (sEPSCs); Spontaneous Inhibitory Postsynaptic Currents (sIPSCs); Spontaneous Recurrent Seizures (SRS); Status Epilepticus (SE); Structure-Activity Relationships (SAR); Superoxide Dismutase (SOD); Synaptic Vesicle Protein 2A (SV2A); Temporal Lobe Epilepsy (TLE); Thiobarbituric Acid Reactive Substances (TBARS); Thyrotropin-Releasing Hormone (TRH); Traumatic Brain Injury (TBI); Tumor Necrosis Factor α (TNF- α); Tumor Protein 53 (Tp53); Tumoral Growth Factor-1 β (TGF-1β); U.S. Food and Drug Administration (FDA); Vascular Endothelial Growth Factor (VEGF); World Health Organization (WHO).

## References

[1] World Health Organization (WHO) Epilepsy. https://www.who.int/news-room/fact-sheets/detail/epilepsy.

[2] Fisher R.S., Acevedo C., Arzimanoglou A., Bogacz A., Cross J.H., Elger C.E., Engel J.J., Forsgren L., French J.A., Glynn M. (2014). ILAE official report: A practical clinical definition of epilepsy. Epilepsia.

[3] Fisher R.S., Cross J.H., French J.A., Higurashi N., Hirsch E., Jansen F.E., Lagae L., Moshé S.L., Peltola J., Roulet Perez E. (2017). Operational classification of seizure types by the International League Against Epilepsy: Position Paper of the ILAE Commission for Classification and Terminology. Epilepsia.

[4] Pérez-Pérez D., Frías-Soria C.L., Rocha L. (2021). Drug-resistant epilepsy: From multiple hypotheses to an integral explanation using preclinical resources. Epilepsy Behav..

[5] Pitkänen A., Sutula T.P. (2002). Is epilepsy a progressive disorder? Prospects for new therapeutic approaches in temporal-lobe epilepsy. Lancet Neurol..

[6] Klein P., Friedman A., Hameed M.Q., Kaminski R.M., Bar-Klein G., Klitgaard H., Koepp M., Jozwiak S., Prince D.A., Rotenberg A. (2020). Repurposed molecules for antiepileptogenesis: Missing an opportunity to prevent epilepsy?. Epilepsia.

[7] Alrabiah H. (2019). Levetiracetam. Profiles Drug Subst. Excip. Relat. Methodol..

[8] Löscher W., Gillard M., Sands Z.A., Kaminski R.M., Klitgaard H. (2016). Synaptic Vesicle Glycoprotein 2A Ligands in the Treatment of Epilepsy and Beyond. CNS Drugs.

[9] Lynch B.A., Lambeng N., Nocka K., Kensel-Hammes P., Bajjalieh S.M., Matagne A., Fuks B. (2004). The synaptic vesicle is the protein SV2A is the binding site for the antiepileptic drug levetiracetam. Proc. Natl. Acad. Sci. USA.

[10] Crepeau A.Z., Treiman D.M. (2010). Levetiracetam: A comprehensive review. Expert Rev. Neurother..

[11] Cortes-Altamirano J.L., Olmos-Hernández A., Bonilla-Jaime H., Bandala C., González-Maciel A., Alfaro-Rodríguez A. (2016). Levetiracetam as an antiepileptic, neuroprotective, and hyperalgesic drug. Neurol. India.

[12] Wong L.C., Freeburg J.D., Montouris G.D., Hohler A.D. (2015). Two patients with Hashimoto’s encephalopathy and uncontrolled diabetes successfully treated with levetiracetam. J. Neurol. Sci..

[13] Rossi S., Mataluni G., Codecà C., Fiore S., Buttari F., Musella A., Castelli M., Bernardi G., Centonze D. (2009). Effects of levetiracetam on chronic pain in multiple sclerosis: Results of a pilot, randomized, placebo-controlled study. Eur. J. Neurol..

[14] Falah M., Madsen C., Holbech J.V., Sindrup S.H. (2012). A randomized, placebo-controlled trial of levetiracetam in central pain in multiple sclerosis. Eur. J. Pain.

[15] Steinhoff B.J., Staack A.M. (2019). Levetiracetam and brivaracetam: A review of evidence from clinical trials and clinical experience. Ther. Adv. Neurol. Disord..

[16] Kenda B.M., Matagne A.C., Talaga P.E., Pasau P.M., Differding E., Lallemand B.I., Frycia A.M., Moureau F.G., Klitgaard H.V., Gillard M.R. (2004). Discovery of 4-substituted pyrrolidone butanamides as new agents with significant antiepileptic activity. J. Med. Chem..

[17] Leclercq K., Matagne A., Provins L., Klitgaard H., Kaminski R.M. (2020). Pharmacological Profile of the Novel Antiepileptic Drug Candidate Padsevonil: Characterization in Rodent Seizure and Epilepsy Models. J. Pharmacol. Exp. Ther..

[18] Surges R., Volynski K.E., Walker M.C. (2008). Is levetiracetam different from other antiepileptic drugs? Levetiracetam and its cellular mechanism of action in epilepsy revisited. Ther. Adv. Neurol. Disord..

[19] Vogl C., Mochida S., Wolff C., Whalley B.J., Stephens G.J. (2012). The synaptic vesicle glycoprotein 2A ligand levetiracetam inhibits presynaptic Ca2+ channels through an intracellular pathway. Mol. Pharmacol..

[20] Bonnet U., Bingmann D., Speckmann E.-J., Wiemann M. (2019). Levetiracetam mediates subtle pH-shifts in adult human neocortical pyramidal cells via an inhibition of the bicarbonate-driven neuronal pH-regulation—Implications for excitability and plasticity modulation. Brain Res..

[21] Lévesque M., Behr C., Avoli M. (2015). The anti-ictogenic effects of levetiracetam are mirrored by interictal spiking and high-frequency oscillation changes in a model of temporal lobe epilepsy. Seizure.

[22] Itoh K., Inamine M., Oshima W., Kotani M., Chiba Y., Ueno M., Ishihara Y. (2015). Prevention of status epilepticus-induced brain edema and neuronal cell loss by repeated treatment with high-dose levetiracetam. Brain Res..

[23] Itoh K., Taniguchi R., Matsuo T., Oguro A., Vogel C.F.A., Yamazaki T., Ishihara Y. (2019). Suppressive effects of levetiracetam on neuroinflammation and phagocytic microglia: A comparative study of levetiracetam, valproate and carbamazepine. Neurosci. Lett..

[24] Sarangi S.C., Pattnaik S.S., Katyal J., Kaleekal T., Dinda A.K. (2020). An interaction study of Ocimum sanctum L. and levetiracetam in pentylenetetrazole kindling model of epilepsy. J. Ethnopharmacol..

[25] Löscher W. (2020). The holy grail of epilepsy prevention: Preclinical approaches to antiepileptogenic treatments. Neuropharmacology.

[26] Hakami T. (2021). Neuropharmacology of Antiseizure Drugs. Neuropsychopharmacol. Rep..

[27] U.S. Food and Drug Administration (FDA) FDA-Approved Drugs. https://www.accessdata.fda.gov/scripts/cder/daf/index.cfm?event=overview.process&ApplNo=021035.

[28] U.S. Food and Drug Administration (FDA) FDA-Approved Drugs. https://www.accessdata.fda.gov/scripts/cder/daf/index.cfm?event=overview.process&ApplNo=021872.

[29] European Medicines Agency (EMA) Keppra. https://www.ema.europa.eu/en/medicines/human/EPAR/keppra#authorisation-details-section.

[30] Italiano D., Ferlazzo E., Gasparini S., Spina E., Mondello S., Labate A., Gambardella A., Aguglia U. (2014). Generalized versus partial reflex seizures: A review. Seizure.

[31] Auvin S. (2008). Treatment of juvenile myoclonic epilepsy. CNS Neurosci. Ther..

[32] Cereghino J.J., Biton V., Abou-Khalil B., Dreifuss F., Gauer L.J., Leppik I. (2000). Levetiracetam for partial seizures: Results of a double-blind, randomized clinical trial. Neurology.

[33] Abou-Khalil B. (2008). Levetiracetam in the treatment of epilepsy. Neuropsychiatr. Dis. Treat..

[34] Shorvon S.D., Löwenthal A., Janz D., Bielen E., Loiseau P. (2000). Multicenter double-blind, randomized, placebo-controlled trial of levetiracetam as add-on therapy in patients with refractory partial seizures. European Levetiracetam Study Group. Epilepsia.

[35] Ben-Menachem E., Falter U. (2000). Efficacy and tolerability of levetiracetam 3000 mg/d in patients with refractory partial seizures: A multicenter, double-blind, responder-selected study evaluating monotherapy. European Levetiracetam Study Group. Epilepsia.

[36] Abdelmesih S.K., Elkhateeb N., Zakaria M., Girgis M.Y. (2021). Initial levetiracetam versus valproate monotherapy in antiseizure medicine (ASM)-naïve pediatric patients with idiopathic generalized epilepsy with tonic-clonic seizures. Seizure.

[37] Falsaperla R., Scalia B., Giugno A., Pavone P., Motta M., Caccamo M., Ruggieri M. (2021). Treating the symptom or treating the disease in neonatal seizures: A systematic review of the literature. Ital. J. Pediatrics.

[38] Özalkaya E., Topcuoglu S., Karatepe H., Tüten A., Gokmen T., Karatekin G. (2019). Efficacy of levetiracetam in premature infants: Our experience and review of the literature. J. Matern.-Fetal Neonatal Med..

[39] Hughes K., Garrity L., Nelson A.S., Lane A., Teusink-Cross A. (2021). Comparison of levetiracetam versus phenytoin/fosphenytoin for busulfan seizure prophylaxis at a pediatric institution. Pediatric Transplant..

[40] Glauser T.A., Pellock J.M., Bebin E.M., Fountain N.B., Ritter F.J., Jensen C.M., Shields W.D. (2002). Efficacy and safety of levetiracetam in children with partial seizures: An open-label trial. Epilepsia.

[41] Piña-Garza J.E., Nordli D.R.J., Rating D., Yang H., Schiemann-Delgado J., Duncan B. (2009). Adjunctive levetiracetam in infants and young children with refractory partial-onset seizures. Epilepsia.

[42] Fayyazi A., Ebrahimi M.H., Roshanaei G., Bazmamoun H. (2021). Evaluation of the Levetiracetam treatment on reduction of epileptic discharges in electroencephalogram in children with epilepsy. Iran. J. Child Neurol..

[43] Trinka E., Cock H., Hesdorffer D., Rossetti A.O., Scheffer I.E., Shinnar S., Shorvon S., Lowenstein D.H. (2015). A definition and classification of status epilepticus--Report of the ILAE Task Force on Classification of Status Epilepticus. Epilepsia.

[44] Chu S.-S., Wang H.-J., Zhu L.-N., Xu D., Wang X.-P., Liu L. (2020). Therapeutic effect of intravenous levetiracetam in status epilepticus: A meta-analysis and systematic review. Seizure.

[45] Yang L., Dong X.-Z., Cui X.-H., Liu J.-M., Liu W.-N., Zhang L. (2021). Comparison of the efficacy and safety of levetiracetam and phenytoin in the treatment of established status epilepticus: A systematic review and meta-analysis. J. Clin. Neurosci. Off. J. Neurosurg. Soc. Australas..

[46] Feng Y., Chen Y., Jia Y., Wang Z., Wang X., Jiang L., Ai C., Li W., Liu Y. (2021). Efficacy and safety of levetiracetam versus (fos)phenytoin for second-line treatment of epilepticus: A meta-analysis of latest randomized controlled trials. Seizure.

[47] Haller J.T., Bonnin S., Radosevich J. (2021). Rapid administration of undiluted intravenous levetiracetam. Epilepsia.

[48] Chen D., Bian H., Zhang L. (2019). A meta-analysis of levetiracetam for randomized placebo-controlled trials in patients with refractory epilepsy. Neuropsychiatr. Dis. Treat..

[49] Qiao M.-Y., Cui H.-T., Zhao L.-Z., Miao J.-K., Chen Q.-X. (2021). Efficacy and Safety of Levetiracetam vs. Phenobarbital for Neonatal Seizures: A Systematic Review and Meta-Analysis. Front. Neurol..

[50] Ruangritkul P., Tiamkao S., Chainirun N., Pranboon S., Tiamkao S., Sawanyawisuth K., Khamsai S. (2021). The Efficacy and Safety Profile of Generic Intravenous Levetiracetam in a Real-World Setting. Curr. Ther. Res. Clin. Exp..

[51] Wright C., Downing J., Mungall D., Khan O., Williams A., Fonkem E., Garrett D., Aceves J., Kirmani B. (2013). Clinical pharmacology and pharmacokinetics of levetiracetam. Front. Neurol..

[52] Hnaini M., Darwich M., Koleilat N., Jaafar F., Hanneyan S., Rahal S., El Mikati I., Shbarou R.M., Nabout R., Maalouf F.I. (2020). High-Dose Levetiracetam for Neonatal Seizures: A Retrospective Review. Seizure.

[53] Besli G.E., Yuksel Karatoprak E., Yilmaz S. (2020). Efficacy and safety profile of intravenous levetiracetam versus phenytoin in convulsive status epilepticus and acute repetitive seizures in children. Epilepsy Behav..

[54] Yi Z.-M., Wen C., Cai T., Xu L., Zhong X.-L., Zhan S.-Y., Zhai S.-D. (2019). Levetiracetam for epilepsy: An evidence map of efficacy, safety and economic profiles. Neuropsychiatr. Dis. Treat..

[55] Verrotti A., Prezioso G., Di Sabatino F., Franco V., Chiarelli F., Zaccara G. (2015). The adverse event profile of levetiracetam: A meta-analysis on children and adults. Seizure.

[56] Chen B., Choi H., Hirsch L.J., Katz A., Legge A., Buchsbaum R., Detyniecki K. (2017). Psychiatric and behavioral side effects of antiepileptic drugs in adults with epilepsy. Epilepsy Behav..

[57] Cortes C., Manterola C. (2020). Behavioral alterations associated with levetiracetam in pediatric epilepsy. Epilepsy Behav..

[58] Kawai M., Goji H., Kanemoto K. (2022). Differences in aggression as psychiatric side effect of levetiracetam and perampanel in patients with epilepsy. Epilepsy Behav..

[59] Moon J.U., Han J.Y. (2021). Comparative Efficacy of Levetiracetam for Epilepsy in School-Aged Children with Intellectual Disability and Normal Intelligence. Brain Sci..

[60] Liu B.-K., Jiang L., Li X.-J., Hong S.-Q., Chen W., Hu Y. (2020). Efficacy and safety of levetiracetam in the off-label treatment of neonatal seizures. Int. J. Neurosci..

[61] Bangash O., Simonin A., Tsimiklis C., Ramakonar H., Honeybul S. (2020). Prophylactic levetiracetam-induced pancytopenia with traumatic extra-dural hematoma: Case report. J. Clin. Neurosci. Off. J. Neurosurg. Soc. Australas..

[62] Fagan A., Fuld J., Soon E. (2017). Levetiracetam-induced eosinophilic pneumonia. BMJ Case Rep..

[63] Gayatri P., Selvam M.M., Sreeharsha S.V. (2020). Levetiracetam-Induced Hepatic Dysfunction. Neurol. India.

[64] Moinuddin I.A. (2020). Suspected Levetiracetam-Induced Rhabdomyolysis: A Case Report and Literature Review. Am. J. Case Rep..

[65] Spencer D. (2017). Levetiracetam in Men With Epilepsy: Testosterone Is Left Alone But Sperm Count Is Paramount. Epilepsy Curr..

[66] Brighina F., Palermo A., Aloisio A., Francolini M., Giglia G., Fierro B. (2006). Levetiracetam in the prophylaxis of migraine with aura: A 6-month open-label study. Clin. Neuropharmacol..

[67] Hamza M.S., Anderson D.G., Snyder J.W., Deschner S., Cifu D.X. (2009). Effectiveness of levetiracetam in the treatment of lumbar radiculopathy: An open-label prospective cohort study. PMR.

[68] Awaad Y., Michon A.M., Minarik S. (2005). Use of levetiracetam to treat tics in children and adolescents with Tourette syndrome. Mov. Disord..

[69] Solaro C., de Sire A., Messmer Uccelli M., Mueller M., Bergamaschi R., Gasperini C., Restivo D.A., Stabile M.R., Patti F. (2020). Efficacy of levetiracetam on upper limb movement in multiple sclerosis patients with cerebellar signs: A multicenter double-blind, placebo-controlled, crossover study. Eur. J. Neurol..

[70] Solaro C., Brichetto G., Capello E., Abuarqub S., Sanguineti V. (2008). Activity, tolerability and efficacy of levetiracetam on cerebellar symptoms in multiple sclerosis patients: A pilot kinematic study. Eur. J. Neurol..

[71] D’Amelio M., Callari G., Gammino M., Saia V., Lupo I., Salemi G., Ragonese P., Savettieri G. (2005). Levetiracetam in the treatment of vascular chorea: A case report. Eur. J. Clin. Pharmacol..

[72] Direk M., Epcacan S., Epcacan Z., Yildirim D.D., Okuyaz C. (2020). Efficacy of levetiracetam in the treatment of Sydenham chorea. Pediatrics Int..

[73] Şahin S., Cansu A. (2015). A New Alternative Drug With Fewer Adverse Effects in the Treatment of Sydenham Chorea: Levetiracetam Efficacy in a Child. Clin. Neuropharmacol..

[74] Wang M., Jiang L., Tang X. (2017). Levetiracetam is associated with decrease in subclinical epileptiform discharges and improved cognitive functions in pediatric patients with autism spectrum disorder. Neuropsychiatr. Dis. Treat..

[75] Deriaz N., Willi J.P., Orihuela-Flores M., Galli Carminati G., Ratib O. (2012). Treatment with levetiracetam in a patient with pervasive developmental disorders, severe intellectual disability, self-injurious behavior, and seizures: A case report. Neurocase.

[76] Farooq M.U., Bhatt A., Majid A., Gupta R., Khasnis A., Kassab M.Y. (2009). Levetiracetam for managing neurologic and psychiatric disorders. Am. J. Health Pharm. AJHP Off. J. Am. Soc. Health Pharm..

[77] Mattes J.A. (2008). Levetiracetam in patients with impulsive aggression: A double-blind, placebo-controlled trial. J. Clin. Psychiatry.

[78] Çökmüş F.P., Aşçıbaşı K., Öztekin S., Demet M.M. (2017). Relationship of levetiracetam and obsessive-compulsive disorder: A case report. Psychiatry Clin. Psychopharmacol..

[79] Esang M., Santos M.G., Ahmed S. (2020). Levetiracetam and Suicidality: A Case Report and Literature Review. Prim. Care Compan. CNS Disord..

[80] Müller C.A., Schäfer M., Schneider S., Heimann H.M., Hinzpeter A., Volkmar K., Förg A., Heinz A., Hein J. (2010). Efficacy and safety of levetiracetam for outpatient alcohol detoxification. Pharmacopsychiatry.

[81] Jabbarli R., Ahmadipour Y., Rauschenbach L., Santos A.N., Darkwah Oppong M., Pierscianek D., Quesada C.M., Kebir S., Dammann P., Guberina N. (2021). How about Levetiracetam in Glioblastoma? An Institutional Experience and Meta-Analysis. Cancers.

[82] Woods S.W., Saksa J.R., Baker C.B., Cohen S.J., Tek C. (2008). Effects of levetiracetam on tardive dyskinesia: A randomized, double-blind, placebo-controlled study. J. Clin. Psychiatry.

[83] Kakisaka Y., Jin K., Fujikawa M., Kitazawa Y., Kato K., Nakasato N. (2017). Levetiracetam improves symptoms of multiple chemical sensitivity: Case report. J. Med. Investig..

[84] Meena N., Satia M.P.S. (2016). A study of the obstetric and perinatal outcomes of eclampsia and the use of levetiracetam in its management. Int. J. Reprod. Contracept. Obs. Gynecol..

[85] Grüter T., Ayzenberg I., Gold R., Börnke C. (2016). Charles Bonnet syndrome successfully treated with levetiracetam. J. Neurol..

[86] Hejazi R.A., Reddymasu S.C., Namin F., Lavenbarg T., Foran P., McCallum R.W. (2010). Efficacy of tricyclic antidepressant therapy in adults with cyclic vomiting syndrome: A two-year follow-up study. J. Clin. Gastroenterol..

[87] Clouse R.E., Sayuk G.S., Lustman P.J., Prakash C. (2007). Zonisamide or levetiracetam for adults with cyclic vomiting syndrome: A case series. Clin. Gastroenterol. Hepatol. Off. Clin. Pract. J. Am. Gastroenterol. Assoc..

[88] Gower A.J., Noyer M., Verloes R., Gobert J., Wülfert E. (1992). ucb L059, a novel anti-convulsant drug: Pharmacological profile in animals. Eur. J. Pharmacol..

[89] Gualtieri F., Manetti D., Romanelli M.N., Ghelardini C. (2002). Design and study of piracetam-like nootropics, controversial members of the problematic class of cognition-enhancing drugs. Curr. Pharm. Des..

[90] Frycia A., Starck J.P., Jadot S., Lallemand B., Leclercq K., Brutto P.L., Matagne A., Verbois V., Mercier J., Kenda B. (2010). Discovery of indolone acetamides as novel SV2A ligands with improved potency toward seizure suppression. ChemMedChem.

[91] Daniels V., Wood M., Leclercq K., Kaminski R.M., Gillard M. (2013). Modulation of the conformational state of the SV2A protein by an allosteric mechanism as evidenced by ligand binding assays. Br. J. Pharmacol..

[92] Bennett B., Matagne A., Michel P., Leonard M., Cornet M., Meeus M.-A., Toublanc N. (2007). Seletracetam (UCB 44212). Neurotherapeutics.

[93] Pollard J.R. (2008). Seletracetam, a small molecule SV2A modulator for the treatment of epilepsy. Curr. Opin. Investig. Drugs.

[94] Malykh A.G., Sadaie M.R. (2010). Piracetam and piracetam-like drugs: From basic science to novel clinical applications to CNS disorders. Drugs.

[95] Abram M., Rapacz A., Mogilski S., Latacz G., Lubelska A., Kamiński R.M., Kamiński K. (2020). Multitargeted Compounds Derived from (2,5-Dioxopyrrolidin-1-yl)(phenyl)-Acetamides as Candidates for Effective Anticonvulsant and Antinociceptive Agents. ACS Chem. Neurosci..

[96] Niespodziany I., Ghisdal P., Mullier B., Wood M., Provins L., Kaminski R.M., Wolff C. (2020). Functional characterization of the antiepileptic drug candidate, padsevonil, on GABA(A) receptors. Epilepsia.

[97] Wood M., Daniels V., Provins L., Wolff C., Kaminski R.M., Gillard M. (2020). Pharmacological Profile of the Novel Antiepileptic Drug Candidate Padsevonil: Interactions with Synaptic Vesicle 2 Proteins and the GABA(A) Receptor. J. Pharmacol. Exp. Ther..

[98] UCB. Chrome-extension://efaidnbmnnnibpcajpcglclefindmkaj/viewer.html?pdfurl=https%3A%2F%2Fwww.ucb.com%2Fsites%2Fdefault%2Ffiles%2Fpress_files%2Fa1e5de61474c0bbf.pdf&clen=513698&chunk=true.

[99] Noyer M., Gillard M., Matagne A., Hénichart J.P., Wülfert E. (1995). The novel antiepileptic drug levetiracetam (ucb L059) appears to act via a specific binding site in CNS membranes. Eur. J. Pharmacol..

[100] Gillard M., Fuks B., Michel P., Vertongen P., Massingham R., Chatelain P. (2003). Binding characteristics of [^3^H]ucb 30889 to levetiracetam binding sites in rat brain. Eur. J. Pharmacol..

[101] Rogawski M.A. (2008). Brivaracetam: A rational drug discovery success story. Br. J. Pharmacol..

[102] Bajjalieh S.M., Peterson K., Shinghal R., Scheller R.H. (1992). SV2, a brain synaptic vesicle protein homologous to bacterial transporters. Science.

[103] Gillard M., Fuks B., Leclercq K., Matagne A. (2011). Binding characteristics of brivaracetam, a selective, high affinity SV2A ligand in rat, mouse and human brain: Relationship to anti-convulsant properties. Eur. J. Pharmacol..

[104] Shi J., Anderson D., Lynch B.A., Castaigne J.-G., Foerch P., Lebon F. (2011). Combining modelling and mutagenesis studies of synaptic vesicle protein 2A to identify a series of residues involved in racetam binding. Biochem. Soc. Trans..

[105] Lee J., Daniels V., Sands Z.A., Lebon F., Shi J., Biggin P.C. (2015). Exploring the interaction of SV2A with racetams using homology modelling, molecular dynamics and site-directed mutagenesis. PLoS ONE.

[106] Correa-Basurto J., Cuevas-Hernández R.I., Phillips-Farfán B.V., Martínez-Archundia M., Romo-Mancillas A., Ramírez-Salinas G.L., Pérez-González Ó.A., Trujillo-Ferrara J., Mendoza-Torreblanca J.G. (2015). Identification of the antiepileptic racetam binding site in the synaptic vesicle protein 2A by molecular dynamics and docking simulations. Front. Cell. Neurosci..

[107] Wood M.D., Gillard M. (2017). Evidence for a differential interaction of brivaracetam and levetiracetam with the synaptic vesicle 2A protein. Epilepsia.

[108] Wood M.D., Sands Z.A., Vandenplas C., Gillard M. (2018). Further evidence for a differential interaction of brivaracetam and levetiracetam with the synaptic vesicle 2A protein. Epilepsia.

[109] Janz R., Goda Y., Geppert M., Missler M., Südhof T.C. (1999). SV2A and SV2B function as redundant Ca2+ regulators in neurotransmitter release. Neuron.

[110] Chang W.-P., Südhof T.C. (2009). SV2 renders primed synaptic vesicles competent for Ca2+ -induced exocytosis. J. Neurosci..

[111] Pichardo L.A., Contreras I.J., Zamudio S.R., Mixcoha E., Mendoza J.G., Talevi A., Rocha L. (2016). Synaptic Vesicle Protein 2A as a novel pharmacological target with broad potential for new antiepileptic drugs. Antiepileptic Drug Discovery: Novel Approaches, Methods in Pharmacology and Toxicology.

[112] Crowder K.M., Gunther J.M., Jones T.A., Hale B.D., Zhang H.Z., Peterson M.R., Scheller R.H., Chavkin C., Bajjalieh S.M. (1999). Abnormal neurotransmission in mice lacking synaptic vesicle protein 2A (SV2A). Proc. Natl. Acad. Sci. USA.

[113] Custer K.L., Austin N.S., Sullivan J.M., Bajjalieh S.M. (2006). Synaptic vesicle protein 2 enhances release probability at quiescent synapses. J. Neurosci..

[114] Vogl C., Tanifuji S., Danis B., Daniels V., Foerch P., Wolff C., Whalley B.J., Mochida S., Stephens G.J. (2015). Synaptic vesicle glycoprotein 2A modulates vesicular release and calcium channel function at peripheral sympathetic synapses. Eur. J. Neurosci..

[115] Xu T., Bajjalieh S.M. (2001). SV2 modulates the size of the readily releasable pool of secretory vesicles. Nat. Cell Biol..

[116] Venkatesan K., Alix P., Marquet A., Doupagne M., Niespodziany I., Rogister B., Seutin V. (2012). Altered balance between excitatory and inhibitory inputs onto CA1 pyramidal neurons from SV2A-deficient but not SV2B-deficient mice. J. Neurosci. Res..

[117] Schivell A.E., Mochida S., Kensel-Hammes P., Custer K.L., Bajjalieh S.M. (2005). SV2A and SV2C contain a unique synaptotagmin-binding site. Mol. Cell. Neurosci..

[118] Yao J., Nowack A., Kensel-Hammes P., Gardner R.G., Bajjalieh S.M. (2010). Cotrafficking of SV2 and synaptotagmin at the synapse. J. Neurosci..

[119] Stout K.A., Dunn A.R., Hoffman C., Miller G.W. (2019). The Synaptic Vesicle Glycoprotein 2: Structure, Function, and Disease Relevance. ACS Chem. Neurosci..

[120] Ciruelas K., Marcotulli D., Bajjalieh S.M. (2019). Synaptic vesicle protein 2: A multi-faceted regulator of secretion. Semin. Cell Dev. Biol..

[121] Nowack A., Malarkey E.B., Yao J., Bleckert A., Hill J., Bajjalieh S.M. (2011). Levetiracetam reverses synaptic deficits produced by overexpression of SV2A. PLoS ONE.

[122] Meehan A.L., Yang X., McAdams B.D., Yuan L.L., Rothman S.M. (2011). A new mechanism for antiepileptic drug action: Vesicular entry may mediate the effects of levetiracetam. J. Neurophysiol..

[123] Mendoza-Torreblanca J.G., Vanoye-Carlo A., Phillips-Farfán B.V., Carmona-Aparicio L., Gómez-Lira G. (2013). Synaptic vesicle protein 2A: Basic facts and role in synaptic function. Eur. J. Neurosci..

[124] Nicolas J.-M., Hannestad J., Holden D., Kervyn S., Nabulsi N., Tytgat D., Huang Y., Chanteux H., Staelens L., Matagne A. (2016). Brivaracetam, a selective high-affinity synaptic vesicle protein 2A (SV2A) ligand with preclinical evidence of high brain permeability and fast onset of action. Epilepsia.

[125] Doheny H.C., Ratnaraj N., Whittington M.A., Jefferys J.G., Patsalos P.N. (1999). Blood and cerebrospinal fluid pharmacokinetics of the novel anticonvulsant levetiracetam (ucb L059) in the rat. Epilepsy Res..

[126] Patsalos P.N. (2000). Pharmacokinetic profile of levetiracetam: Toward ideal characteristics. Pharmacol. Ther..

[127] Tong X., Patsalos P.N. (2001). A microdialysis study of the novel antiepileptic drug levetiracetam: Extracellular pharmacokinetics and effect on taurine in rat brain. Br. J. Pharmacol..

[128] Kaminski R.M., Gillard M., Leclercq K., Hanon E., Lorent G., Dassesse D., Matagne A., Klitgaard H. (2009). Proepileptic phenotype of SV2A-deficient mice is associated with reduced anticonvulsant efficacy of levetiracetam. Epilepsia.

[129] De Groot M., Aronica E., Heimans J.J., Reijneveld J.C. (2011). Synaptic vesicle protein 2A predicts response to levetiracetam in patients with glioma. Neurology.

[130] Ohno Y., Ishihara S., Terada R., Kikuta M., Sofue N., Kawai Y., Serikawa T., Sasa M. (2009). Preferential increase in the hippocampal synaptic vesicle protein 2A (SV2A) by pentylenetetrazole kindling. Biochem. Biophys. Res. Commun..

[131] Matveeva E.A., Vanaman T.C., Whiteheart S.W., Slevin J.T. (2008). Levetiracetam prevents kindling-induced asymmetric accumulation of hippocampal 7S SNARE complexes. Epilepsia.

[132] Inaba T., Miyamoto N., Hira K., Ueno Y., Yamashiro K., Watanabe M., Shimada Y., Hattori N., Urabe T. (2019). Protective Role of Levetiracetam Against Cognitive Impairment And Brain White Matter Damage in Mouse prolonged Cerebral Hypoperfusion. Neuroscience.

[133] Contreras-García I.J., Gómez-Lira G., Phillips-Farfán B.V., Pichardo-Macías L.A., García-Cruz M.E., Chávez-Pacheco J.L., Mendoza-Torreblanca J.G. (2021). Synaptic Vesicle Protein 2A Expression in Glutamatergic Terminals Is Associated with the Response to Levetiracetam Treatment. Brain Sci..

[134] Marcotulli D., Fattorini G., Bragina L., Perugini J., Conti F. (2017). Levetiracetam Affects Differentially Presynaptic Proteins in Rat Cerebral Cortex. Front. Cell. Neurosci..

[135] Niespodziany I., Klitgaard H., Margineanu D.G. (2001). Levetiracetam inhibits the high-voltage-activated Ca(2+) current in pyramidal neurones of rat hippocampal slices. Neurosci. Lett..

[136] Costa C., Martella G., Picconi B., Prosperetti C., Pisani A., Di Filippo M., Pisani F., Bernardi G., Calabresi P. (2006). Multiple mechanisms underlying the neuroprotective effects of antiepileptic drugs against in vitro ischemia. Stroke.

[137] Pisani A., Bonsi P., Martella G., De Persis C., Costa C., Pisani F., Bernardi G., Calabresi P. (2004). Intracellular calcium increase in epileptiform activity: Modulation by levetiracetam and lamotrigine. Epilepsia.

[138] Lukyanetz E.A., Shkryl V.M., Kostyuk P.G. (2002). Selective blockade of N-type calcium channels by levetiracetam. Epilepsia.

[139] Yan H.-D., Ishihara K., Seki T., Hanaya R., Kurisu K., Arita K., Serikawa T., Sasa M. (2013). Inhibitory effects of levetiracetam on the high-voltage-activated L-type Ca^2+^ channels in hippocampal CA3 neurons of spontaneously epileptic rat (SER). Brain Res. Bull..

[140] Deshpande L.S., Delorenzo R.J. (2014). Mechanisms of levetiracetam in the control of status epilepticus and epilepsy. Front. Neurol..

[141] Angehagen M., Margineanu D.G., Ben-Menachem E., Rönnbäck L., Hansson E., Klitgaard H. (2003). Levetiracetam reduces caffeine-induced Ca2+ transients and epileptiform potentials in hippocampal neurons. Neuroreport.

[142] Nagarkatti N., Deshpande L.S., DeLorenzo R.J. (2008). Levetiracetam inhibits both ryanodine and IP3 receptor activated calcium induced calcium release in hippocampal neurons in culture. Neurosci. Lett..

[143] Cataldi M., Lariccia V., Secondo A., di Renzo G., Annunziato L. (2005). The antiepileptic drug levetiracetam decreases the inositol 1,4,5-trisphosphate-dependent [Ca2+]I increase induced by ATP and bradykinin in PC12 cells. J. Pharmacol. Exp. Ther..

[144] Navidhamidi M., Ghasemi M., Mehranfard N. (2017). Epilepsy-associated alterations in hippocampal excitability. Rev. Neurosci..

[145] Rigo J.-M., Hans G., Nguyen L., Rocher V., Belachew S., Malgrange B., Leprince P., Moonen G., Selak I., Matagne A. (2002). The anti-epileptic drug levetiracetam reverses the inhibition by negative allosteric modulators of neuronal GABA- and glycine-gated currents. Br. J. Pharmacol..

[146] Doelken M.T., Hammen T., Bogner W., Mennecke A., Stadlbauer A., Boettcher U., Doerfler A., Stefan H. (2010). Alterations of intracerebral γ-aminobutyric acid (GABA) levels by titration with levetiracetam in patients with focal epilepsies. Epilepsia.

[147] Li Q., Chen C., Gong T. (2018). High-field MRS study of GABA+ in patients with migraine: Response to levetiracetam treatment. Neuroreport.

[148] Klitgaard H., Matagne A., Grimee R., Vanneste-Goemaere J., Margineanu D.G. (2003). Electrophysiological, neurochemical and regional effects of levetiracetam in the rat pilocarpine model of temporal lobe epilepsy. Seizure.

[149] Fukuyama K., Tanahashi S., Nakagawa M., Yamamura S., Motomura E., Shiroyama T., Tanii H., Okada M. (2012). Levetiracetam inhibits neurotransmitter release associated with CICR. Neurosci. Lett..

[150] Pichardo Macías L.A., Ramírez Mendiola B.A., Contreras García I.J., Zamudio Hernández S.R., Chávez Pacheco J.L., Sánchez Huerta K.B., Mendoza Torreblanca J.G. (2018). Effect of levetiracetam on extracellular amino acid levels in the dorsal hippocampus of rats with temporal lobe epilepsy. Epilepsy Res..

[151] Löscher W., Hönack D., Bloms-Funke P. (1996). The novel antiepileptic drug levetiracetam (ucb L059) induces alterations in GABA metabolism and turnover in discrete areas of rat brain and reduces neuronal activity in substantia nigra pars reticulata. Brain Res..

[152] Mazzuferi M., Palma E., Martinello K., Maiolino F., Roseti C., Fucile S., Fabene P.F., Schio F., Pellitteri M., Sperk G. (2010). Enhancement of GABA(A)-current run-down in the hippocampus occurs at the first spontaneous seizure in a model of temporal lobe epilepsy. Proc. Natl. Acad. Sci. USA.

[153] Cifelli P., Palma E., Roseti C., Verlengia G., Simonato M. (2013). Changes in the sensitivity of GABAA current rundown to drug treatments in a model of temporal lobe epilepsy. Front. Cell. Neurosci..

[154] Palma E., Ragozzino D., Di Angelantonio S., Mascia A., Maiolino F., Manfredi M., Cantore G., Esposito V., Di Gennaro G., Quarato P. (2007). The antiepileptic drug levetiracetam stabilizes the human epileptic GABAA receptors upon repetitive activation. Epilepsia.

[155] Malatynska E., Knapp R., Ikeda M., Yamamura H.I. (1989). Beta-carboline interactions at the BZ-GABA receptor chloride-ionophore complex in the rat cerebral cortex. Brain Res. Bull..

[156] Evans A.K., Lowry C.A. (2007). Pharmacology of the beta-carboline FG-7,142, a partial inverse agonist at the benzodiazepine allosteric site of the GABA A receptor: Neurochemical, neurophysiological, and behavioral effects. CNS Drug Rev..

[157] Kulick C.V., Gutherz S.B., Beck V.C., Medvedeva N., Soper C., Forcelli P.A. (2014). Profile of anticonvulsant action of levetiracetam, tiagabine and phenobarbital against seizures evoked by DMCM (methyl-6,7-dimethoxy-4-ethyl-β-carboline-3-carboxylate) in neonatal rats. Eur. J. Pharmacol..

[158] Wakita M., Kotani N., Kogure K., Akaike N. (2014). Inhibition of excitatory synaptic transmission in hippocampal neurons by levetiracetam involves Zn^2+^-dependent GABA type A receptor-mediated presynaptic modulation. J. Pharmacol. Exp. Ther..

[159] Buckley K., Kelly R.B. (1985). Identification of a transmembrane glycoprotein specific for secretory vesicles of neural and endocrine cells. J. Cell Biol..

[160] Bajjalieh S.M., Frantz G.D., Weimann J.M., McConnell S.K., Scheller R.H. (1994). Differential expression of synaptic vesicle protein 2 (SV2) isoforms. J. Neurosci..

[161] Bragina L., Fattorini G., Giovedí S., Melone M., Bosco F., Benfenati F., Conti F. (2011). Analysis of Synaptotagmin, SV2, and Rab3 Expression in Cortical Glutamatergic and GABAergic Axon Terminals. Front. Cell. Neurosci..

[162] Grønborg M., Pavlos N.J., Brunk I., Chua J.J.E., Münster-Wandowski A., Riedel D., Ahnert-Hilger G., Urlaub H., Jahn R. (2010). Quantitative comparison of glutamatergic and GABAergic synaptic vesicles unveils selectivity for few proteins including MAL2, a novel synaptic vesicle protein. J. Neurosci..

[163] Tokudome K., Okumura T., Terada R., Shimizu S., Kunisawa N., Mashimo T., Serikawa T., Sasa M., Ohno Y. (2016). A Missense Mutation of the Gene Encoding Synaptic Vesicle Glycoprotein 2A (SV2A) Confers Seizure Susceptibility by Disrupting Amygdalar Synaptic GABA Release. Front. Pharmacol..

[164] Ohno Y., Tokudome K. (2017). Therapeutic Role of Synaptic Vesicle Glycoprotein 2A (SV2A) in Modulating Epileptogenesis. CNS Neurol. Disord. Drug Targets.

[165] Contreras-García I.J., Pichardo-Macías L.A., Santana-Gómez C.E., Sánchez-Huerta K., Ramírez-Hernández R., Gómez-González B., Rocha L., Mendoza Torreblanca J.G. (2018). Differential expression of synaptic vesicle protein 2A after status epilepticus and during epilepsy in a lithium-pilocarpine model. Epilepsy Behav..

[166] Tokudome K., Okumura T., Shimizu S., Mashimo T., Takizawa A., Serikawa T., Terada R., Ishihara S., Kunisawa N., Sasa M. (2016). Synaptic vesicle glycoprotein 2A (SV2A) regulates kindling epileptogenesis via GABAergic neurotransmission. Sci. Rep..

[167] Mendoza-Torreblanca J.G., García-Cruz M.E., Sánchez-Cruz I., Gomez-Gonzalez B., Juárez-Méndez S., Gómez-Lira G. (2019). Analysis of Differential Expression of Synaptic Vesicle Protein 2A in the Adult Rat Brain. Neuroscience.

[168] Micov A., Tomić M., Popović B., Stepanović-Petrović R. (2010). The antihyperalgesic effect of levetiracetam in an inflammatory model of pain in rats: Mechanism of action. Br. J. Pharmacol..

[169] Stepanović-Petrović R.M., Micov A.M., Tomić M.A., Ugrešić N.D. (2012). The local peripheral antihyperalgesic effect of levetiracetam and its mechanism of action in an inflammatory pain model. Anesth. Analg..

[170] Carunchio I., Pieri M., Ciotti M.T., Albo F., Zona C. (2007). Modulation of AMPA receptors in cultured cortical neurons induced by the antiepileptic drug levetiracetam. Epilepsia.

[171] Hentschke M., Wiemann M., Hentschke S., Kurth I., Hermans-Borgmeyer I., Seidenbecher T., Jentsch T.J., Gal A., Hübner C.A. (2006). Mice with a targeted disruption of the Cl-/HCO3- exchanger AE3 display a reduced seizure threshold. Mol. Cell. Biol..

[172] Svichar N., Esquenazi S., Chen H.-Y., Chesler M. (2011). Preemptive regulation of intracellular pH in hippocampal neurons by a dual mechanism of depolarization-induced alkalinization. J. Neurosci..

[173] Sander T., Toliat M.R., Heils A., Leschik G., Becker C., Rüschendorf F., Rohde K., Mundlos S., Nürnberg P. (2002). Association of the 867Asp variant of the human anion exchanger 3 gene with common subtypes of idiopathic generalized epilepsy. Epilepsy Res..

[174] Leniger T., Thöne J., Bonnet U., Hufnagel A., Bingmann D., Wiemann M. (2004). Levetiracetam inhibits Na+-dependent Cl-/HCO3- exchange of adult hippocampal CA3 neurons from guinea-pigs. Br. J. Pharmacol..

[175] Gu J., Lynch B.A., Anderson D., Klitgaard H., Lu S., Elashoff M., Ebert U., Potschka H., Löscher W. (2004). The antiepileptic drug levetiracetam selectively modifies kindling-induced alterations in gene expression in the temporal lobe of rats. Eur. J. Neurosci..

[176] Husum H., Bolwig T.G., Sánchez C., Mathé A.A., Hansen S.L. (2004). Levetiracetam prevents changes in levels of brain-derived neurotrophic factor and neuropeptide Y mRNA and of Y1- and Y5-like receptors in the hippocampus of rats undergoing amygdala kindling: Implications for antiepileptogenic and mood-stabilizing proper. Epilepsy Behav..

[177] Christensen K.V., Leffers H., Watson W.P., Sánchez C., Kallunki P., Egebjerg J. (2010). Levetiracetam attenuates hippocampal expression of synaptic plasticity-related immediate early and late response genes in amygdala-kindled rats. BMC Neurosci..

[178] Kim J.-E., Choi H.-C., Song H.-K., Jo S.-M., Kim D.-S., Choi S.-Y., Kim Y.-I., Kang T.-C. (2010). Levetiracetam inhibits interleukin-1 beta inflammatory responses in the hippocampus and piriform cortex of epileptic rats. Neurosci. Lett..

[179] Rassu M., Biosa A., Galioto M., Fais M., Sini P., Greggio E., Piccoli G., Crosio C., Iaccarino C. (2019). Levetiracetam treatment ameliorates LRRK2 pathological mutant phenotype. J. Cell. Mol. Med..

[180] Kovacevic J., Maroteaux G., Schut D., Loos M., Dubey M., Pitsch J., Remmelink E., Koopmans B., Crowley J., Cornelisse L.N. (2018). Protein instability, haploinsufficiency, and cortical hyper-excitability underlie STXBP1 encephalopathy. Brain.

[181] Dilena R., Striano P., Traverso M., Viri M., Cristofori G., Tadini L., Barbieri S., Romeo A., Zara F. (2016). Dramatic effect of levetiracetam in early-onset epileptic encephalopathy due to STXBP1 mutation. Brain Dev..

[182] Parveen B., Tripathi M., Vohora D. (2018). A Cross-Sectional Study to Assess the Modulation of Wnt Inhibitors following Anti-Epileptic Drug Therapy and their Correlation with Vitamin D and Receptor Activator of Nuclear Factor κ B Ligand in Indian Women with Epilepsy. Basic Clin. Pharmacol. Toxicol..

[183] Lange F., Weßlau K., Porath K., Hörnschemeyer J., Bergner C., Krause B.J., Mullins C.S., Linnebacher M., Köhling R., Kirschstein T. (2019). AMPA receptor antagonist perampanel affects glioblastoma cell growth and glutamate release in vitro. PLoS ONE.

[184] Niidome K., Taniguchi R., Yamazaki T., Tsuji M., Itoh K., Ishihara Y. (2021). FosL1 Is a Novel Target of Levetiracetam for Suppressing the Microglial Inflammatory Reaction. Int. J. Mol. Sci..

[185] Hassel B., Taubøll E., Shaw R., Gjerstad L., Dingledine R. (2010). Region-specific changes in gene expression in rat brain after chronic treatment with levetiracetam or phenytoin. Epilepsia.

[186] Sills G.J., Rogawski M.A. (2020). Mechanisms of action of currently used antiseizure drugs. Neuropharmacology.

[187] Belcastro V., Pierguidi L., Tambasco N. (2011). Levetiracetam in brain ischemia: Clinical implications in neuroprotection and prevention of post-stroke epilepsy. Brain Dev..

[188] Rakhade S.N., Shah A.K., Agarwal R., Yao B., Asano E., Loeb J.A. (2007). Activity-dependent gene expression correlates with interictal spiking in human neocortical epilepsy. Epilepsia.

[189] Arion D., Sabatini M., Unger T., Pastor J., Alonso-Nanclares L., Ballesteros-Yáñez I., García Sola R., Muñoz A., Mirnics K., DeFelipe J. (2006). Correlation of transcriptome profile with electrical activity in temporal lobe epilepsy. Neurobiol. Dis..

[190] Margineanu D.G., Matagne A., Kaminski R.M., Klitgaard H. (2008). Effects of chronic treatment with levetiracetam on hippocampal field responses after pilocarpine-induced status epilepticus in rats. Brain Res. Bull..

[191] Zhao T., Yu J., Wang T.-T., Feng J., Zhao W.-B., Sun L., Yu L.-H., Li H.-J., Sun Y. (2020). Impact of ABCB1 Polymorphism on Levetiracetam Serum Concentrations in Epileptic Uygur Children in China. Ther. Drug Monit..

[192] Calame D.G., Herman I., Riviello J.J. (2021). A de novo heterozygous rare variant in SV2A causes epilepsy and levetiracetam-induced drug-resistant status epilepticus. Epilepsy Behav. Rep..

[193] Wolking S., Moreau C., Nies A.T., Schaeffeler E., McCormack M., Auce P., Avbersek A., Becker F., Krenn M., Møller R.S. (2020). Testing association of rare genetic variants with resistance to three common antiseizure medications. Epilepsia.

[194] Grimminger T., Pernhorst K., Surges R., Niehusmann P., Priebe L., von Lehe M., Hoffmann P., Cichon S., Schoch S., Becker A.J. (2013). Levetiracetam resistance: Synaptic signatures & corresponding promoter SNPs in epileptic hippocampi. Neurobiol. Dis..

[195] Helmstaedter C., Mihov Y., Toliat M.R., Thiele H., Nuernberg P., Schoch S., Surges R., Elger C.E., Kunz W.S., Hurlemann R. (2013). Genetic variation in dopaminergic activity is associated with the risk for psychiatric side effects of levetiracetam. Epilepsia.

[196] Ulloa C.M., Towfigh A., Safdieh J. (2009). Review of levetiracetam, with a focus on the extended release formulation, as adjuvant therapy in controlling partial-onset seizures. Neuropsychiatr. Dis. Treat..

[197] Löscher W., Hönack D. (1993). Differences in anticonvulsant potency and adverse effects between dextromethorphan and dextrorphan in amygdala-kindled and non-kindled rats. Eur. J. Pharmacol..

[198] Klitgaard H., Matagne A., Gobert J., Wülfert E. (1998). Evidence for a unique profile of levetiracetam in rodent models of seizures and epilepsy. Eur. J. Pharmacol..

[199] Kupferberg H. (2001). Animal models used in the screening of antiepileptic drugs. Epilepsia.

[200] Klitgaard H. (2001). Levetiracetam: The preclinical profile of a new class of antiepileptic drugs?. Epilepsia.

[201] Birnstiel S., Wülfert E., Beck S.G. (1997). Levetiracetam (ucb LO59) affects in vitro models of epilepsy in CA3 pyramidal neurons without altering normal synaptic transmission. Naunyn-Schmiedeberg’s Arch. Pharmacol..

[202] Morgan O., Medenwald B. (2020). Safety and Tolerability of Rapid Administration Undiluted Levetiracetam. Neurocrit. Care.

[203] Glien M., Brandt C., Potschka H., Löscher W. (2002). Effects of the novel antiepileptic drug levetiracetam on spontaneous recurrent seizures in the rat pilocarpine model of temporal lobe epilepsy. Epilepsia.

[204] Ji-Qun C., Ishihara K., Nagayama T., Serikawa T., Sasa M. (2005). Long-lasting antiepileptic effects of levetiracetam against epileptic seizures in the spontaneously epileptic rat (SER): Differentiation of levetiracetam from conventional antiepileptic drugs. Epilepsia.

[205] Oliveira A.A., Nogueira C.R.A., Nascimento V.S., Aguiar L.M.V., Freitas R.M., Sousa F.C.F., Viana G.S.B., Fonteles M.M.F. (2005). Evaluation of levetiracetam effects on pilocarpine-induced seizures: Cholinergic muscarinic system involvement. Neurosci. Lett..

[206] Löscher W., Hönack D. (2000). Development of tolerance during chronic treatment of kindled rats with the novel antiepileptic drug levetiracetam. Epilepsia.

[207] Song H., Tufa U., Chow J., Sivanenthiran N., Cheng C., Lim S., Wu C., Feng J., Eubanks J.H., Zhang L. (2018). Effects of Antiepileptic Drugs on Spontaneous Recurrent Seizures in a Novel Model of Extended Hippocampal Kindling in Mice. Front. Pharmacol..

[208] Doheny H.C., Whittington M.A., Jefferys J.G.R., Patsalos P.N. (2002). A comparison of the efficacy of carbamazepine and the novel anti-epileptic drug levetiracetam in the tetanus toxin model of focal complex partial epilepsy. Br. J. Pharmacol..

[209] Gower A.J., Hirsch E., Boehrer A., Noyer M., Marescaux C. (1995). Effects of levetiracetam, a novel antiepileptic drug, on convulsant activity in two genetic rat models of epilepsy. Epilepsy Res..

[210] Bouwman B.M., van Rijn C.M. (2004). Effects of levetiracetam on spike and wave discharges in WAG/Rij rats. Seizure.

[211] Talos D.M., Chang M., Kosaras B., Fitzgerald E., Murphy A., Folkerth R.D., Jensen F.E. (2013). Antiepileptic effects of levetiracetam in a rodent neonatal seizure model. Pediatric Res..

[212] van Vliet E.A., van Schaik R., Edelbroek P.M., da Silva F.H.L., Wadman W.J., Gorter J.A. (2008). Development of tolerance to levetiracetam in rats with chronic epilepsy. Epilepsia.

[213] Zheng Y., Moussally J., Cash S.S., Karnam H.B., Cole A.J. (2010). Intravenous levetiracetam in the rat pilocarpine-induced status epilepticus model: Behavioral, physiological and histological studies. Neuropharmacology.

[214] ClinicalTrials.gov U.S. National Library of Medicine. www.clinicaltrials.gov.

[215] Stodieck S., Steinhoff B.J., Kolmsee S., van Rijckevorsel K. (2001). Effect of levetiracetam in patients with epilepsy and interictal epileptiform discharges. Seizure.

[216] Yang X.F., Weisenfeld A., Rothman S.M. (2007). Prolonged exposure to levetiracetam reveals a presynaptic effect on neurotransmission. Epilepsia.

[217] Lee C.-Y., Chen C.-C., Liou H.-H. (2009). Levetiracetam inhibits glutamate transmission through presynaptic P/Q-type calcium channels on the granule cells of the dentate gyrus. Br. J. Pharmacol..

[218] Löscher W., Hönack D., Rundfeldt C. (1998). Antiepileptogenic effects of the novel anticonvulsant levetiracetam (ucb L059) in the kindling model of temporal lobe epilepsy. J. Pharmacol. Exp. Ther..

[219] Stratton S.C., Large C.H., Cox B., Davies G., Hagan R.M. (2003). Effects of lamotrigine and levetiracetam on seizure development in a rat amygdala kindling model. Epilepsy Res..

[220] Vinogradova L.V., van Rijn C.M. (2008). Anticonvulsive and antiepileptogenic effects of levetiracetam in the audiogenic kindling model. Epilepsia.

[221] Yan H.-D., Ji-qun C., Ishihara K., Nagayama T., Serikawa T., Sasa M. (2005). Separation of antiepileptogenic and antiseizure effects of levetiracetam in the spontaneously epileptic rat (SER). Epilepsia.

[222] Sugaya Y., Jinde S., Kato N., Maru E. (2010). Levetiracetam inhibits kindling-induced synaptic potentiation in the dentate gyrus of freely moving rats. Neurosci. Res..

[223] Leo A., De Caro C., Nesci V., Palma E., Tallarico M., Iannone M., Constanti A., De Sarro G., Russo E., Citraro R. (2019). Antiepileptogenic effects of Ethosuximide and Levetiracetam in WAG/Rij rats are only temporary. Pharmacol. Rep..

[224] Sugaya Y., Maru E., Kudo K., Shibasaki T., Kato N. (2010). Levetiracetam suppresses development of spontaneous EEG seizures and aberrant neurogenesis following kainate-induced status epilepticus. Brain Res..

[225] Brandt C., Glien M., Gastens A.M., Fedrowitz M., Bethmann K., Volk H.A., Potschka H., Löscher W. (2007). Prophylactic treatment with levetiracetam after status epilepticus: Lack of effect on epileptogenesis, neuronal damage, and behavioral alterations in rats. Neuropharmacology.

[226] Christensen J., Pedersen M.G., Pedersen C.B., Sidenius P., Olsen J., Vestergaard M. (2009). Long-term risk of epilepsy after traumatic brain injury in children and young adults: A population-based cohort study. Lancet.

[227] Fiani B., Andraos C., Mabry I., Siddiqi J. (2021). A Comparison of Seizure Prophylaxis: Phenytoin Versus Levetiracetam. Cureus.

[228] Fang T., Valdes E., Frontera J.A. (2022). Levetiracetam for Seizure Prophylaxis in Neurocritical Care: A Systematic Review and Meta-analysis. Neurocrit. Care.

[229] Löscher W., Brandt C. (2010). Prevention or modification of epileptogenesis after brain insults: Experimental approaches and translational research. Pharmacol. Rev..

[230] Milligan T.A., Hurwitz S., Bromfield E.B. (2008). Efficacy and tolerability of levetiracetam versus phenytoin after supratentorial neurosurgery. Neurology.

[231] Iuchi T., Kuwabara K., Matsumoto M., Kawasaki K., Hasegawa Y., Sakaida T. (2015). Levetiracetam versus phenytoin for seizure prophylaxis during and early after craniotomy for brain tumours: A phase II prospective, randomised study. J. Neurol. Neurosurg. Psychiatry.

[232] Klein P., Herr D., Pearl P.L., Natale J., Levine Z., Nogay C., Sandoval F., Trzcinski S., Atabaki S.M., Tsuchida T. (2012). Results of phase 2 safety and feasibility study of treatment with levetiracetam for prevention of posttraumatic epilepsy. Arch. Neurol..

[233] Kruer R.M., Harris L.H., Goodwin H., Kornbluth J., Thomas K.P., Slater L.A., Haut E.R. (2013). Changing trends in the use of seizure prophylaxis after traumatic brain injury: A shift from phenytoin to levetiracetam. J. Crit. Care.

[234] Radic J.A.E., Chou S.H.-Y., Du R., Lee J.W. (2014). Levetiracetam versus phenytoin: A comparison of efficacy of seizure prophylaxis and adverse event risk following acute or subacute subdural hematoma diagnosis. Neurocrit. Care.

[235] Falsaperla R., Mauceri L., Pavone P., Barbagallo M., Vitaliti G., Ruggieri M., Pisani F., Corsello G. (2019). Short-Term Neurodevelopmental Outcome in Term Neonates Treated with Phenobarbital versus Levetiracetam: A Single-Center Experience. Behav. Neurol..

[236] Fuller K.L., Wang Y.Y., Cook M.J., Murphy M.A., D’Souza W.J. (2013). Tolerability, safety, and side effects of levetiracetam versus phenytoin in intravenous and total prophylactic regimen among craniotomy patients: A prospective randomized study. Epilepsia.

[237] Taylor S., Heinrichs R.J., Janzen J.M., Ehtisham A. (2011). Levetiracetam is associated with improved cognitive outcome for patients with intracranial hemorrhage. Neurocrit. Care.

[238] Pang X.-M., Liang X.-L., Zhou X., Liu J.-P., Zhang Z., Zheng J.-O. (2020). Alterations in intra- and internetwork functional connectivity associated with levetiracetam treatment in temporal lobe epilepsy. Neurol. Sci. Off. J. Ital. Neurol. Soc. Ital. Soc. Clin. Neurophysiol..

[239] Wandschneider B., Stretton J., Sidhu M., Centeno M., Kozák L.R., Symms M., Thompson P.J., Duncan J.S., Koepp M.J. (2014). Levetiracetam reduces abnormal network activations in temporal lobe epilepsy. Neurology.

[240] Cavarsan C.F., Malheiros J., Hamani C., Najm I., Covolan L. (2018). Is mossy fiber sprouting a potential therapeutic target for epilepsy?. Front. Neurol..

[241] Kutlu G., Gomceli Y.B., Unal Y., Inan L.E. (2008). Levetiracetam monotherapy for late poststroke seizures in the elderly. Epilepsy Behav..

[242] Marini H., Altavilla D., Bellomo M., Adamo E.B., Marini R., Laureanti F., Bonaccorso M.C., Seminara P., Passaniti M., Minutoli L. (2004). Modulation of IL-1 beta gene expression by lipid peroxidation inhibition after kainic acid-induced rat brain injury. Exp. Neurol..

[243] Lee D.-S., Ryu H.J., Kim J.-E., Choi H.-C., Kim Y.-I., Song H.-K., Kang T.-C. (2013). The effect of levetiracetam on status epilepticus-induced neuronal death in the rat hippocampus. Seizure.

[244] Itoh K., Ishihara Y., Komori R., Nochi H., Taniguchi R., Chiba Y., Ueno M., Takata-Tsuji F., Dohgu S., Kataoka Y. (2016). Levetiracetam treatment influences blood-brain barrier failure associated with angiogenesis and inflammatory responses in the acute phase of epileptogenesis in post-status epilepticus mice. Brain Res..

[245] Shetty A.K. (2013). Prospects of levetiracetam as a neuroprotective drug against status epilepticus, traumatic brain injury, and stroke. Front. Neurol..

[246] Gibbs J.E., Walker M.C., Cock H.R. (2006). Levetiracetam: Antiepileptic properties and protective effects on mitochondrial dysfunction in experimental status epilepticus. Epilepsia.

[247] Santana-Gómez C.E., Valle-Dorado M.G., Domínguez-Valentín A.E., Hernández-Moreno A., Orozco-Suárez S., Rocha L. (2018). Neuroprotective effects of levetiracetam, both alone and combined with propylparaben, in the long-term consequences induced by lithium-pilocarpine status epilepticus. Neurochem. Int..

[248] Hanon E., Klitgaard H. (2001). Neuroprotective properties of the novel antiepileptic drug levetiracetam in the rat middle cerebral artery occlusion model of focal cerebral ischemia. Seizure.

[249] Kilicdag H., Daglıoglu K., Erdogan S., Guzel A., Sencar L., Polat S., Zorludemir S. (2013). The effect of levetiracetam on neuronal apoptosis in neonatal rat model of hypoxic ischemic brain injury. Early Hum. Dev..

[250] Yao X., Yang W., Ren Z., Zhang H., Shi D., Li Y., Yu Z., Guo Q., Yang G., Gu Y. (2021). Neuroprotective and Angiogenesis Effects of Levetiracetam Following Ischemic Stroke in Rats. Front. Pharmacol..

[251] Wang H., Gao J., Lassiter T.F., McDonagh D.L., Sheng H., Warner D.S., Lynch J.R., Laskowitz D.T. (2006). Levetiracetam is neuroprotective in murine models of closed head injury and subarachnoid hemorrhage. Neurocrit. Care.

[252] Xiong J., Zhou H., Lu D., Wang Z., Liu H., Sun Y., Xu J., Feng Y., Xing A. (2020). Levetiracetam Reduces Early Inflammatory Response After Experimental Intracerebral Hemorrhage by Regulating the Janus Kinase 2 (JAK2)-Signal Transducer and Activator of Transcription 3 (STAT3) Signaling Pathway. Med. Sci. Monit. Int. Med. J. Exp. Clin. Res..

[253] Mohammad H.M.F., Sami M.M., Makary S., Toraih E.A., Mohamed A.O., El-Ghaiesh S.H. (2019). Neuroprotective effect of levetiracetam in mouse diabetic retinopathy: Effect on glucose transporter-1 and GAP43 expression. Life Sci..

[254] Lima R., Gomes E.D., Cibrão J.R., Rocha L.A., Assunção-Silva R.C., Rodrigues C.S., Neves-Carvalho A., Monteiro S., Salgado A.J., Silva N.A. (2021). Levetiracetam treatment leads to functional recovery after thoracic or cervical injuries of the spinal cord. NPJ Regen. Med..

[255] Bonifacio S.L., Gonzalez F., Ferriero D.M., Christine A.G., Devaskar S.U. (2012). Chapter 61—Central Nervous System Injury and Neuroprotection. Avery’s Diseases of the Newborn (Ninth Edition).

[256] Klitgaard H., Pitkänen A. (2003). Antiepileptogenesis, neuroprotection, and disease modification in the treatment of epilepsy: Focus on levetiracetam. Epileptic Disord..

[257] Calabresi P., Cupini L.M., Centonze D., Pisani F., Bernardi G. (2003). Antiepileptic drugs as a possible neuroprotective strategy in brain ischemia. Ann. Neurol..

[258] Vezzani A., Fujinami R.S., White H.S., Preux P.-M., Blümcke I., Sander J.W., Löscher W. (2016). Infections, inflammation and epilepsy. Acta Neuropathol..

[259] Ambrogini P., Torquato P., Bartolini D., Albertini M.C., Lattanzi D., Di Palma M., Marinelli R., Betti M., Minelli A., Cuppini R. (2019). Excitotoxicity, neuroinflammation and oxidant stress as molecular bases of epileptogenesis and epilepsy-derived neurodegeneration: The role of vitamin E. Biochim. Biophys. Acta. Mol. Basis Dis..

[260] Sanz P., Garcia-Gimeno M.A. (2020). Reactive Glia Inflammatory Signaling Pathways and Epilepsy. Int. J. Mol. Sci..

[261] Vrinda M., Arun S., Srikumar B.N., Kutty B.M., Shankaranarayana Rao B.S. (2019). Temporal lobe epilepsy-induced neurodegeneration and cognitive deficits: Implications for aging. J. Chem. Neuroanat..

[262] Vezzani A., French J., Bartfai T., Baram T.Z. (2011). The role of inflammation in epilepsy. Nat. Rev. Neurol..

[263] Pracucci E., Pillai V., Lamers D., Parra R., Landi S. (2021). Neuroinflammation: A Signature or a Cause of Epilepsy?. Int. J. Mol. Sci..

[264] Devinsky O., Vezzani A., Najjar S., De Lanerolle N.C., Rogawski M.A. (2013). Glia and epilepsy: Excitability and inflammation. Trends Neurosci..

[265] Behl T., Makkar R., Sehgal A., Singh S., Sharma N., Zengin G., Bungau S., Andronie-Cioara F.L., Munteanu M.A., Brisc M.C. (2021). Current Trends in Neurodegeneration: Cross Talks between Oxidative Stress, Cell Death, and Inflammation. Int. J. Mol. Sci..

[266] Farrell J.S., Wolff M.D., Teskey G.C. (2017). Neurodegeneration and Pathology in Epilepsy: Clinical and Basic Perspectives. Adv. Neurobiol..

[267] Martinc B., Grabnar I., Vovk T. (2014). Antioxidants as a preventive treatment for epileptic process: A review of the current status. Curr. Neuropharmacol..

[268] Beltrán-Sarmiento E., Arregoitia-Sarabia C.K., Floriano-Sánchez E., Sandoval-Pacheco R., Galván-Hernández D.E., Coballase-Urrutia E., Carmona-Aparicio L., Ramos-Reyna E., Rodríguez-Silverio J., Cárdenas-Rodríguez N. (2018). Effects of Valproate Monotherapy on the Oxidant-Antioxidant Status in Mexican Epileptic Children: A Longitudinal Study. Oxid. Med. Cell. Longev..

[269] Nazıroğlu M., Yürekli V.A. (2013). Effects of antiepileptic drugs on antioxidant and oxidant molecular pathways: Focus on trace elements. Cell. Mol. Neurobiol..

[270] Hansson E., Björklund U., Skiöldebrand E., Rönnbäck L. (2018). Anti-inflammatory effects induced by pharmaceutical substances on inflammatory active brain astrocytes-promising treatment of neuroinflammation. J. Neuroinflamm..

[271] Osuntokun O.S., Abdulwahab U.F., Akanji N.O., Adedokun K.I., Adekomi A.D., Olayiwola G. (2021). Anticonvulsant and neuroprotective effects of carbamazepine-levetiracetam adjunctive treatment in convulsive status epilepticus rat model: Inhibition of cholinergic transmission. Neurosci. Lett..

[272] Bayhan I., Turtay M.G., Ciftci O., Cetin A., Basak N., Namık Oztanır M., Oguzturk H., Gurbuz S., Guven T. (2020). Comparison of immunological, histological and oxidative effects of felbamate and levetiracetam in traumatic brain injury. Eur. Rev. Med. Pharmacol. Sci..

[273] de Souza A.G., Chaves Filho A.J.M., Souza Oliveira J.V., de Souza D.A.A., Lopes I.S., de Carvalho M.A.J., de Lima K.A., Florenço Sousa F.C., Mendes Vasconcelos S.M., Macedo D. (2019). Prevention of pentylenetetrazole-induced kindling and behavioral comorbidities in mice by levetiracetam combined with the GLP-1 agonist liraglutide: Involvement of brain antioxidant and BDNF upregulating properties. Biomed. Pharmacother..

[274] Abdel-Wahab B.A., Shaikh I.A., Khateeb M.M., Habeeb S.M. (2015). Omega 3 polyunsaturated fatty acids enhance the protective effect of levetiracetam against seizures, cognitive impairment and hippocampal oxidative DNA damage in young kindled rats. Pharmacol. Biochem. Behav..

[275] Mazhar F., Malhi S.M., Simjee S.U. (2017). Comparative studies on the effects of clinically used anticonvulsants on the oxidative stress biomarkers in pentylenetetrazole-induced kindling model of epileptogenesis in mice. J. Basic Clin. Physiol. Pharmacol..

[276] Imran I., Koch K., Schöfer H., Lau H., Klein J. (2019). Effects of Three Anti-Seizure Drugs on Cholinergic and Metabolic Activity in Experimental Status Epilepticus. J. Pharm. Pharm. Sci. Publ. Can. Soc. Pharm. Sci. Soc. Can. Sci. Pharm..

[277] Oliveira A.A., Almeida J.P.C., Freitas R.M., Nascimento V.S., Aguiar L.M.V., Júnior H.V.N., Fonseca F.N., Viana G.S.B., Sousa F.C.F., Fonteles M.M.F. (2007). Effects of levetiracetam in lipid peroxidation level, nitrite-nitrate formation and antioxidant enzymatic activity in mice brain after pilocarpine-induced seizures. Cell. Mol. Neurobiol..

[278] Dircio-Bautista M., Colín-González A.L., Aguilera G., Maya-López M., Villeda-Hernández J., Galván-Arzate S., García E., Túnez I., Santamaría A. (2018). The Antiepileptic Drug Levetiracetam Protects Against Quinolinic Acid-Induced Toxicity in the Rat Striatum. Neurotox. Res..

[279] Erbaş O., Yılmaz M., Taşkıran D. (2016). Levetiracetam attenuates rotenone-induced toxicity: A rat model of Parkinson’s disease. Environ. Toxicol. Pharmacol..

[280] Erbaş O., Oltulu F., Yılmaz M., Yavaşoğlu A., Taşkıran D. (2016). Neuroprotective effects of chronic administration of levetiracetam in a rat model of diabetic neuropathy. Diabetes Res. Clin. Pract..

[281] Akman L., Erbas O., Akdemir A., Yavasoglu A., Taskiran D., Kazandi M. (2015). Levetiracetam ameliorates ovarian function in streptozotocin-induced diabetic rats. Gynecol. Endocrinol. Off. J. Int. Soc. Gynecol. Endocrinol..

[282] Sarangi S.C., Kakkar A.K., Kumar R., Gupta Y.K. (2016). Effect of lamotrigine, levetiracetam & topiramate on neurobehavioural parameters & oxidative stress in comparison with valproate in rats. Indian J. Med. Res..

[283] Baysal M., Ilgin S., Kilic G., Kilic V., Ucarcan S., Atli O. (2017). Reproductive toxicity after levetiracetam administration in male rats: Evidence for role of hormonal status and oxidative stress. PLoS ONE.

[284] Ersan S., Cigdem B., Bakir D., Dogan H.O. (2020). Determination of levels of oxidative stress and nitrosative stress in patients with epilepsy. Epilepsy Res..

[285] Mahdavi A., Naeini A.A., Najafi M., Maracy M., Ghazvini M.A. (2020). Effect of levetiracetam drug on antioxidant and liver enzymes in epileptic patients: Case-control study. Afr. Health Sci..

[286] Haznedar P., Doğan Ö., Albayrak P., Öz Tunçer G., Teber S., Deda G., Eminoglu F.T. (2019). Effects of levetiracetam and valproic acid treatment on liver function tests, plasma free carnitine and lipid peroxidation in childhood epilepsies. Epilepsy Res..

[287] Ozden H., Kabay S.C., Toker A., Ustüner M.C., Ozbayer C., Ustüner D., Günes H.V. (2010). The effects of levetiracetam on urinary 15f-2t-isoprostane levels in epileptic patients. Seizure.

[288] Varoglu A.O., Yildirim A., Aygul R., Gundogdu O.L., Sahin Y.N. (2010). Effects of valproate, carbamazepine, and levetiracetam on the antioxidant and oxidant systems in epileptic patients and their clinical importance. Clin. Neuropharmacol..

[289] Morimoto M., Hashimoto T., Kitaoka T., Kyotani S. (2018). Impact of Oxidative Stress and Newer Antiepileptic Drugs on the Albumin and Cortisol Value in Severe Motor and Intellectual Disabilities With Epilepsy. J. Clin. Med. Res..

[290] Morimoto M., Satomura S., Hashimoto T., Kyotani S. (2017). A study of oxidative stress and the newer antiepileptic drugs in epilepsy associated with severe motor and intellectual disabilities. J. Chin. Med. Assoc..

[291] Chen W., Tan Y., Ge Y., Chen Y., Liu X. (2015). The Effects of Levetiracetam on Cerebrospinal Fluid and Plasma NPY and GAL, and on the Components of Stress Response System, hs-CRP, and S100B Protein in Serum of Patients with Refractory Epilepsy. Cell Biochem. Biophys..

[292] Stienen M.N., Haghikia A., Dambach H., Thöne J., Wiemann M., Gold R., Chan A., Dermietzel R., Faustmann P.M., Hinkerohe D. (2011). Anti-inflammatory effects of the anticonvulsant drug levetiracetam on electrophysiological properties of astroglia are mediated via TGFβ1 regulation. Br. J. Pharmacol..

[293] Haghikia A., Ladage K., Hinkerohe D., Vollmar P., Heupel K., Dermietzel R., Faustmann P.M. (2008). Implications of antiinflammatory properties of the anticonvulsant drug levetiracetam in astrocytes. J. Neurosci. Res..

[294] Thöne J., Ellrichmann G., Faustmann P.M., Gold R., Haghikia A. (2012). Anti-inflammatory effects of levetiracetam in experimental autoimmune encephalomyelitis. Int. Immunopharmacol..

[295] Guenther S., Bauer S., Hagge M., Knake S., Olmes D.G., Tackenberg B., Rosenow F., Hamer H.M. (2014). Chronic valproate or levetiracetam treatment does not influence cytokine levels in humans. Seizure.

[296] Labh R., Gupta R., Narang M., Halder S., Kar R. (2021). Effect of valproate and add-on levetiracetam on inflammatory biomarkers in children with epilepsy. Epilepsy Behav..

[297] Gulcebi M.I., Kendirli T., Turgan Z.A., Patsalos P.N., Onat Yilmaz F. (2018). The effect of serum levetiracetam concentrations on therapeutic response and IL1-beta concentration in patients with epilepsy. Epilepsy Res..

[298] Stettner M., Dehmel T., Mausberg A.K., Köhne A., Rose C.R., Kieseier B.C. (2011). Levetiracetam exhibits protective properties on rat Schwann cells in vitro. J. Peripher. Nerv. Syst..

[299] Irwin M.H., Moos W.H., Faller D.V., Steliou K., Pinkert C.A. (2016). Epigenetic Treatment of Neurodegenerative Disorders: Alzheimer and Parkinson Diseases. Drug Dev. Res..

[300] Hoffmann A., Kann O., Ohlemeyer C., Hanisch U.-K., Kettenmann H. (2003). Elevation of basal intracellular calcium as a central element in the activation of brain macrophages (microglia): Suppression of receptor-evoked calcium signaling and control of release function. J. Neurosci..

[301] Marciani M.G., Gotman J., Andermann F., Olivier A. (1985). Patterns of Seizure Activation after Withdrawal of Antiepileptic Medication. Neurology.

[302] Costa A.-M., Lucchi C., Malkoç A., Rustichelli C., Biagini G. (2021). Relationship between Delta Rhythm, Seizure Occurrence and Allopregnanolone Hippocampal Levels in Epileptic Rats Exposed to the Rebound Effect. Pharmaceuticals.

[303] Boon P., Chauvel P., Pohlmann-Eden B., Otoul C., Wroe S. (2002). Dose-Response Effect of Levetiracetam 1000 and 2000 Mg/Day in Partial Epilepsy. Epilepsy Res..

[304] Hansen C.C., Ljung H., Brodtkorb E., Reimers A. (2018). Mechanisms Underlying Aggressive Behavior Induced by Antiepileptic Drugs: Focus on Topiramate, Levetiracetam, and Perampanel. Behav. Neurol..

[305] Brodie M.J., Besag F., Ettinger A.B., Mula M., Gobbi G., Comai S., Aldenkamp A.P., Steinhoff B.J. (2016). Epilepsy, Antiepileptic Drugs, and Aggression: An Evidence-Based Review. Pharmacol. Rev..

[306] Steinhoff B.J., Klein P., Klitgaard H., Laloyaux C., Moseley B.D., Ricchetti-Masterson K., Rosenow F., Sirven J.I., Smith B., Stern J.M. (2021). Behavioral Adverse Events with Brivaracetam, Levetiracetam, Perampanel, and Topiramate: A Systematic Review. Epilepsy Behav..

